# Supplementary material for: RNA aptamers specific for transmembrane p24 trafficking protein 6 and Clusterin for the targeted delivery of imaging reagents and RNA therapeutics to human β cells
Source: Nat Commun. 2022 Apr 5;13:1815. doi: 10.1038/s41467-022-29377-3 (PMC8983715; doi:10.1038/s41467-022-29377-3)
Supplement: Supplementary file 1 — Supplementary Information [file 41467_2022_29377_MOESM1_ESM.pdf]

Supplementary materials for

RNA aptamers specific for transmembrane p24 trafficking protein 6 and Clusterin for the targeted delivery of imaging reagents and RNA therapeutics to human β cells

Dimitri Van Simaeys, Adriana De La Fuente, Serena Zilio, Alessia Zoso, Victoria Kuznetsova, Oscar Alcazar, Peter Buchwald, Andrea Grilli, Jimmy Caroli, Silvio Bicciato, and Paolo Serafini*

* * Correspondence should be addressed to P.S. (email: pserafini@miami.edu)

This PDF file includes:

Supplementary figures 1 to 9 and

Supplementary tables 1 to 6

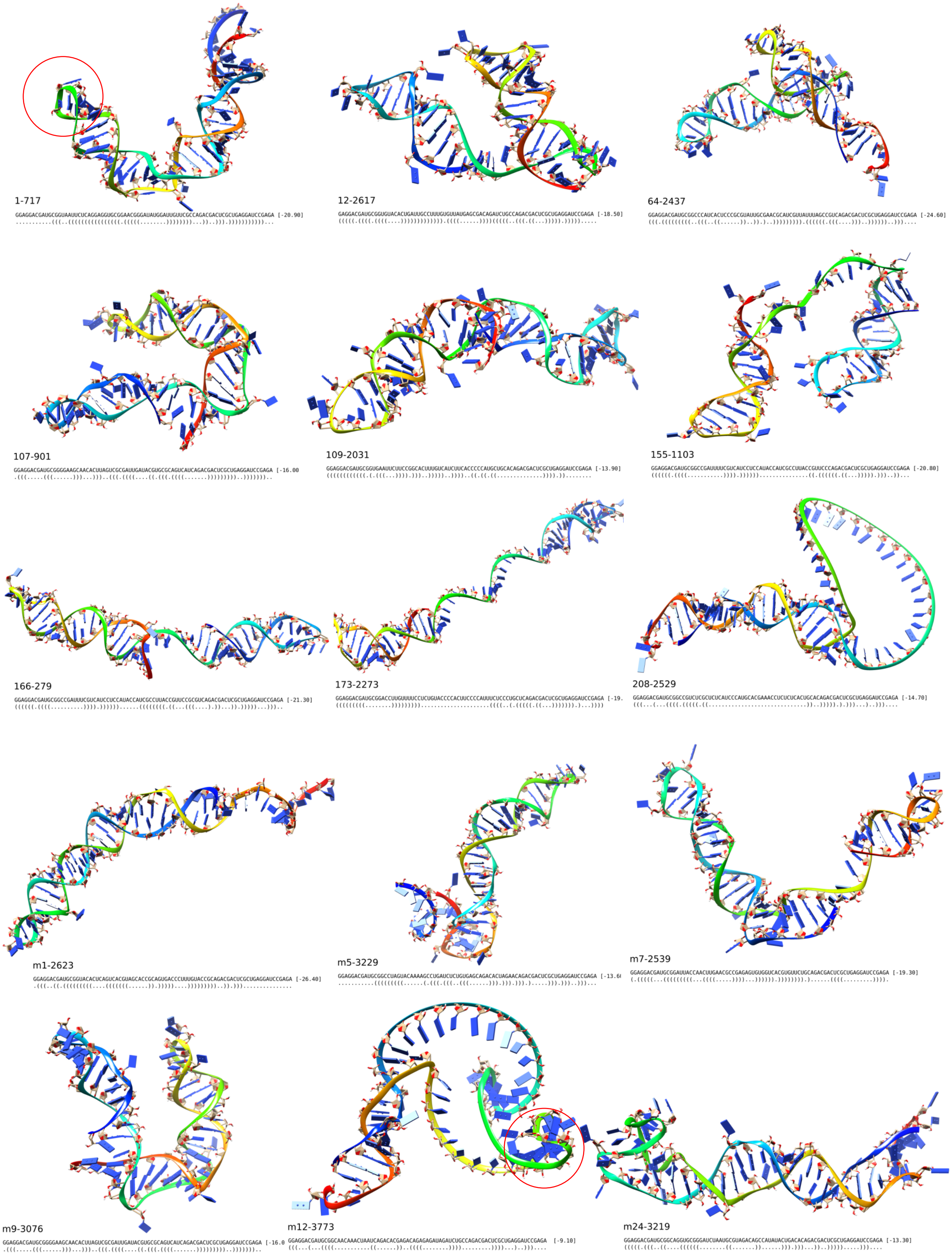

Supplementary Figure 1: 3D structures of the 15 aptamers chosen for empirical validation. Tertiary structures for apt 1-717 and m12-3773 were modeled using RNAComposer (http://rnacomposer.cs.put.poznan.pl/) web modeling server based on the secondary structures and enriched motifs identified by APTANI run on cycle H8 library

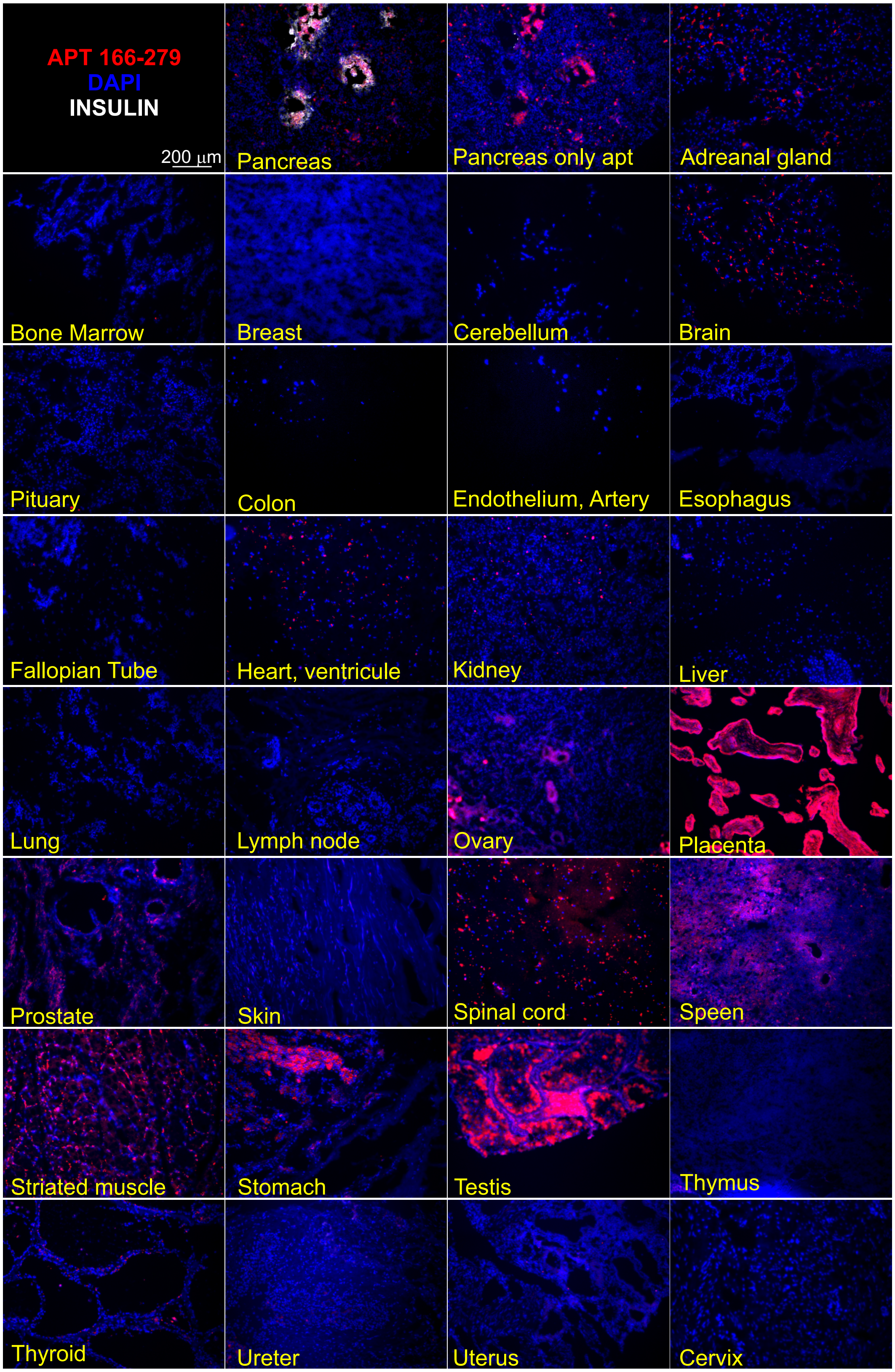

Supplementary figure 2: Aptamer binding to human tissue arrays highlights a reasonable specificity of aptamer 1-717 and m12-3773 for human islets. FDA approved, snap-frozen human tissue arrays were stained with the indicated Cy3 labeled aptamers (red) and counterstained with anti-insulin antibodies (white) and DAPI (blue). Images were acquired with the Zeiss Apotome fluorescence microscope. One representative image of two independent experiments is shown in each panel. **Figure continue in the next pages**

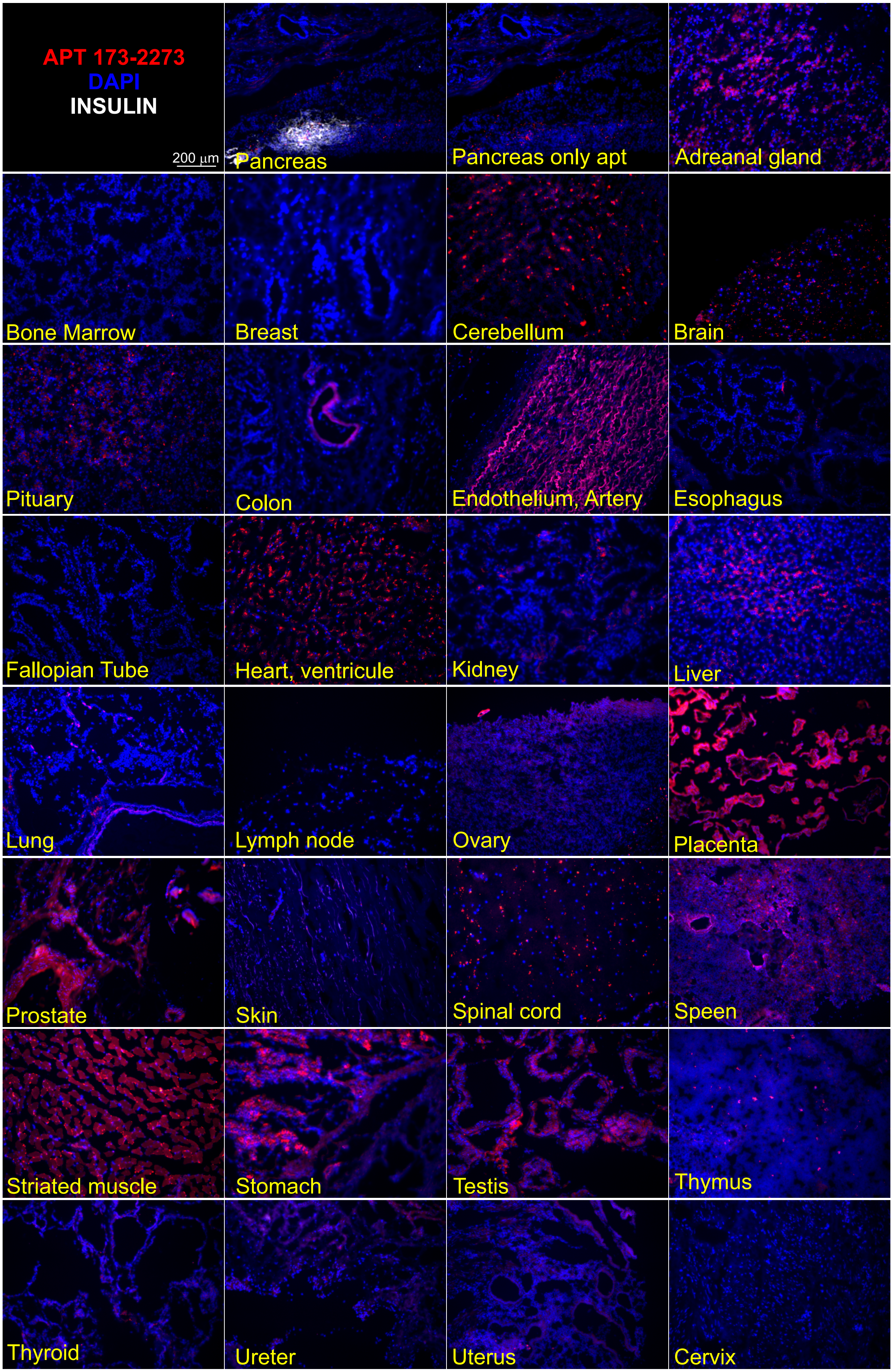

Supplementary figure 2 continued

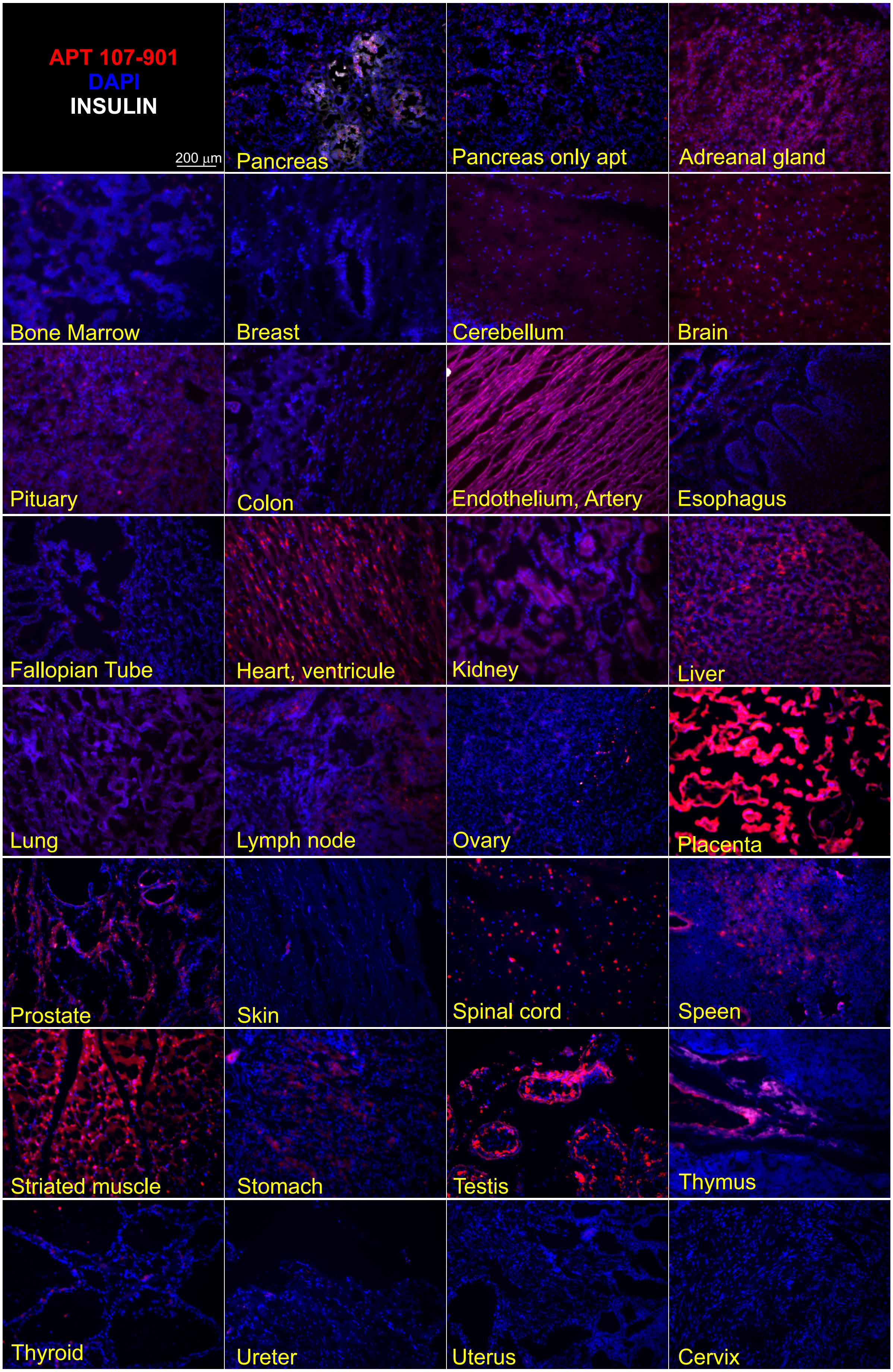

Supplementary figure 2 continued

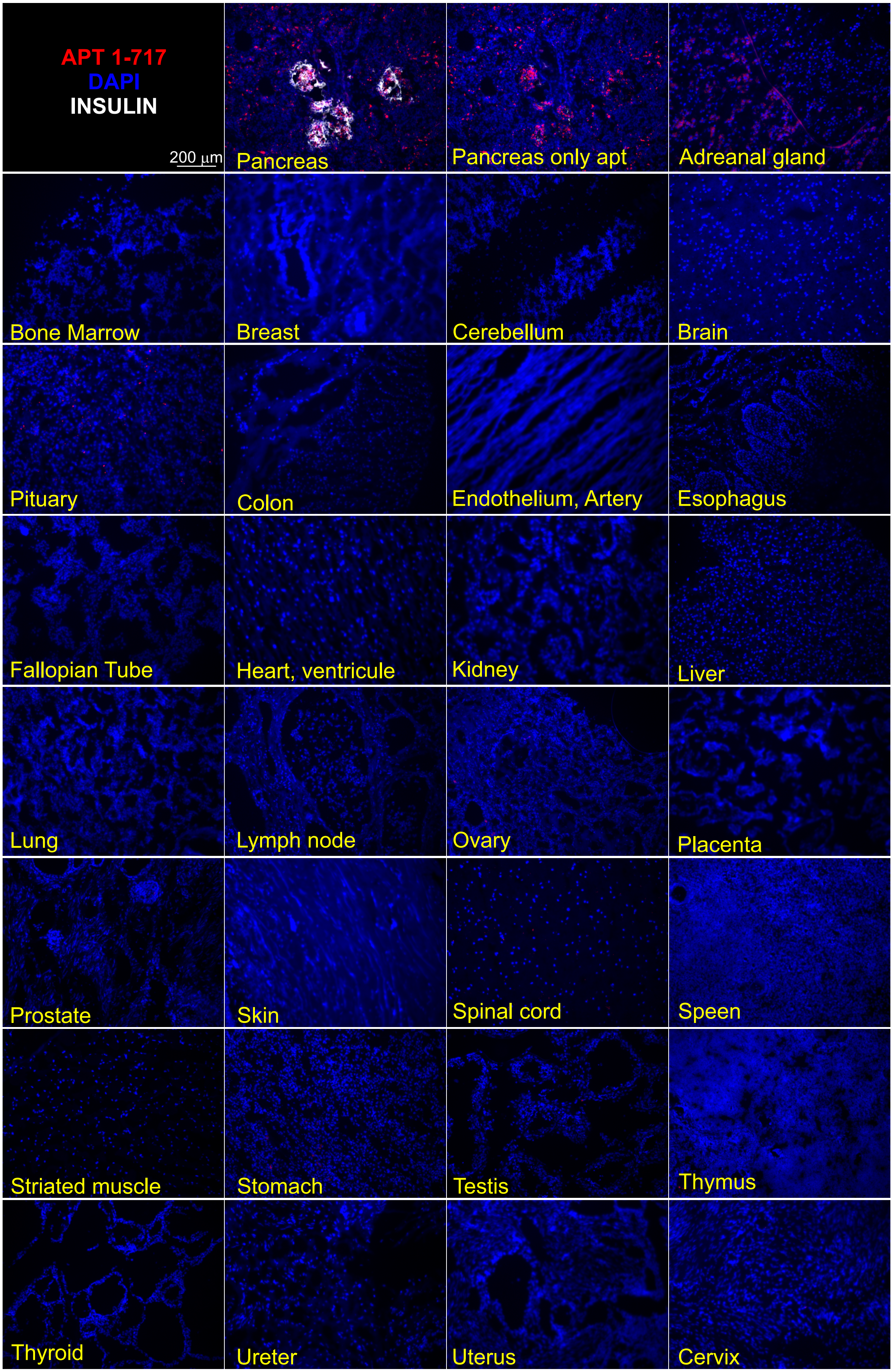

Supplementary figure 2 continued

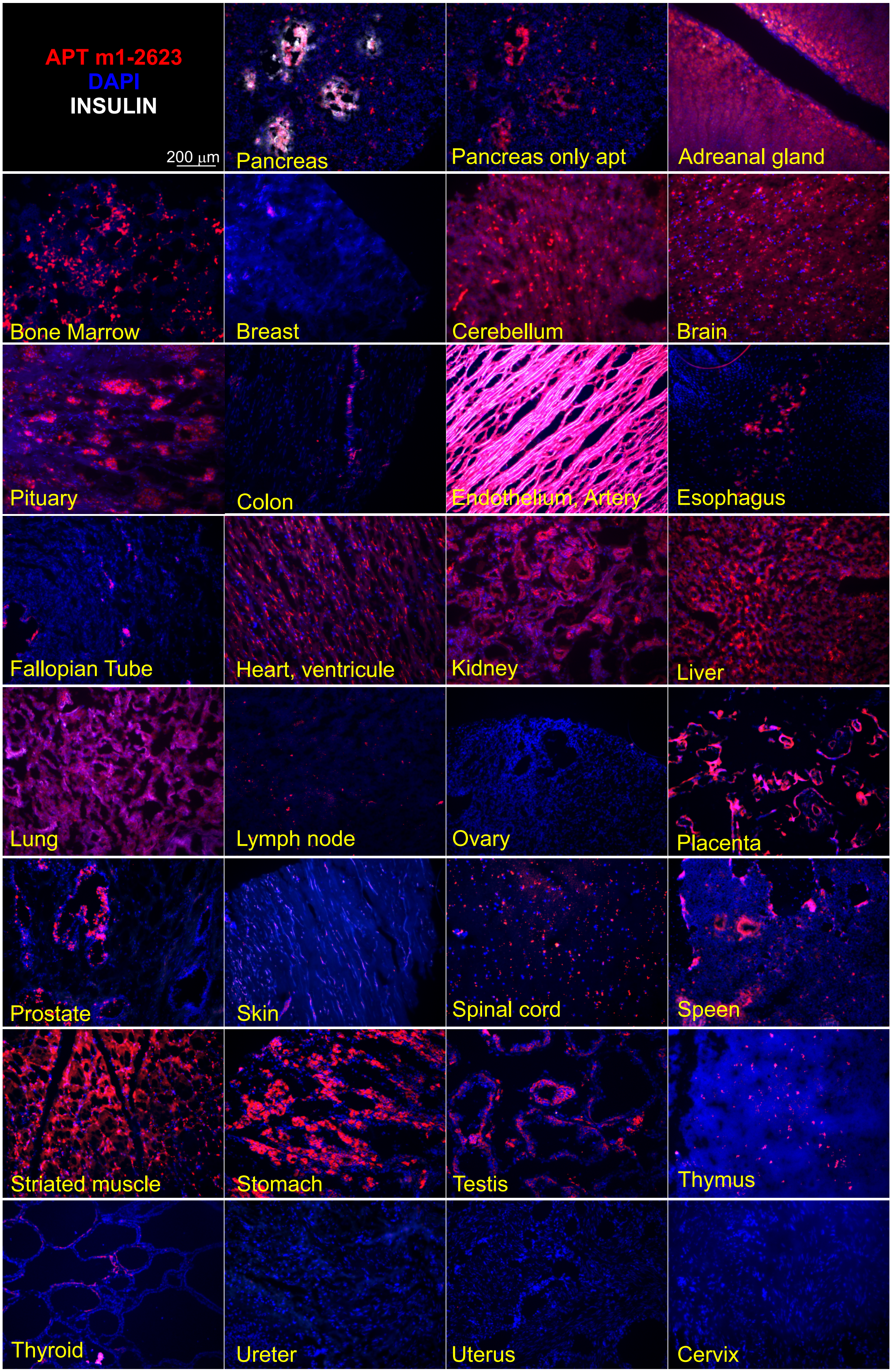

Supplementary figure 2 continued

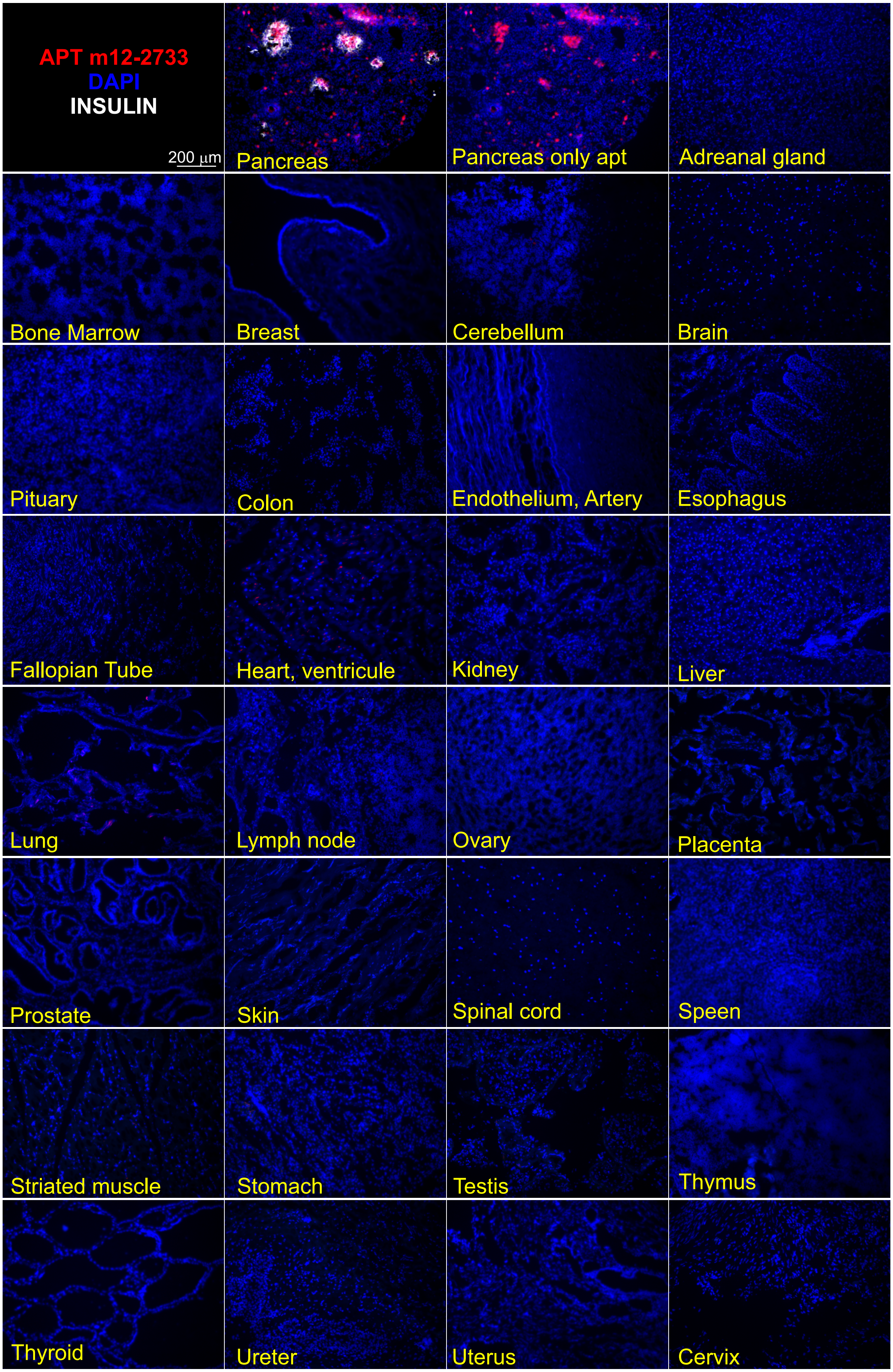

Supplementary figure 2 continued

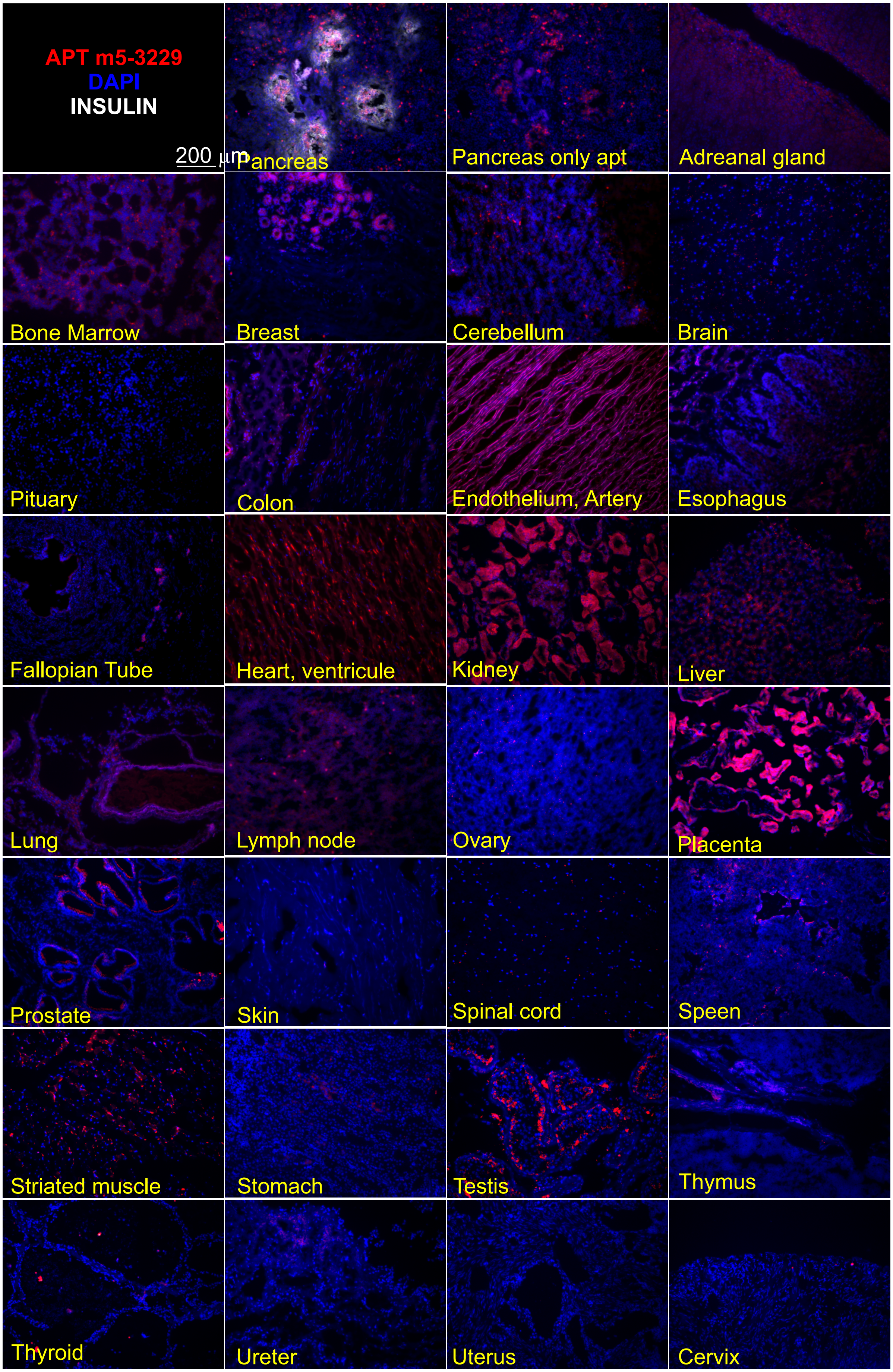

Supplementary figure 2 continued

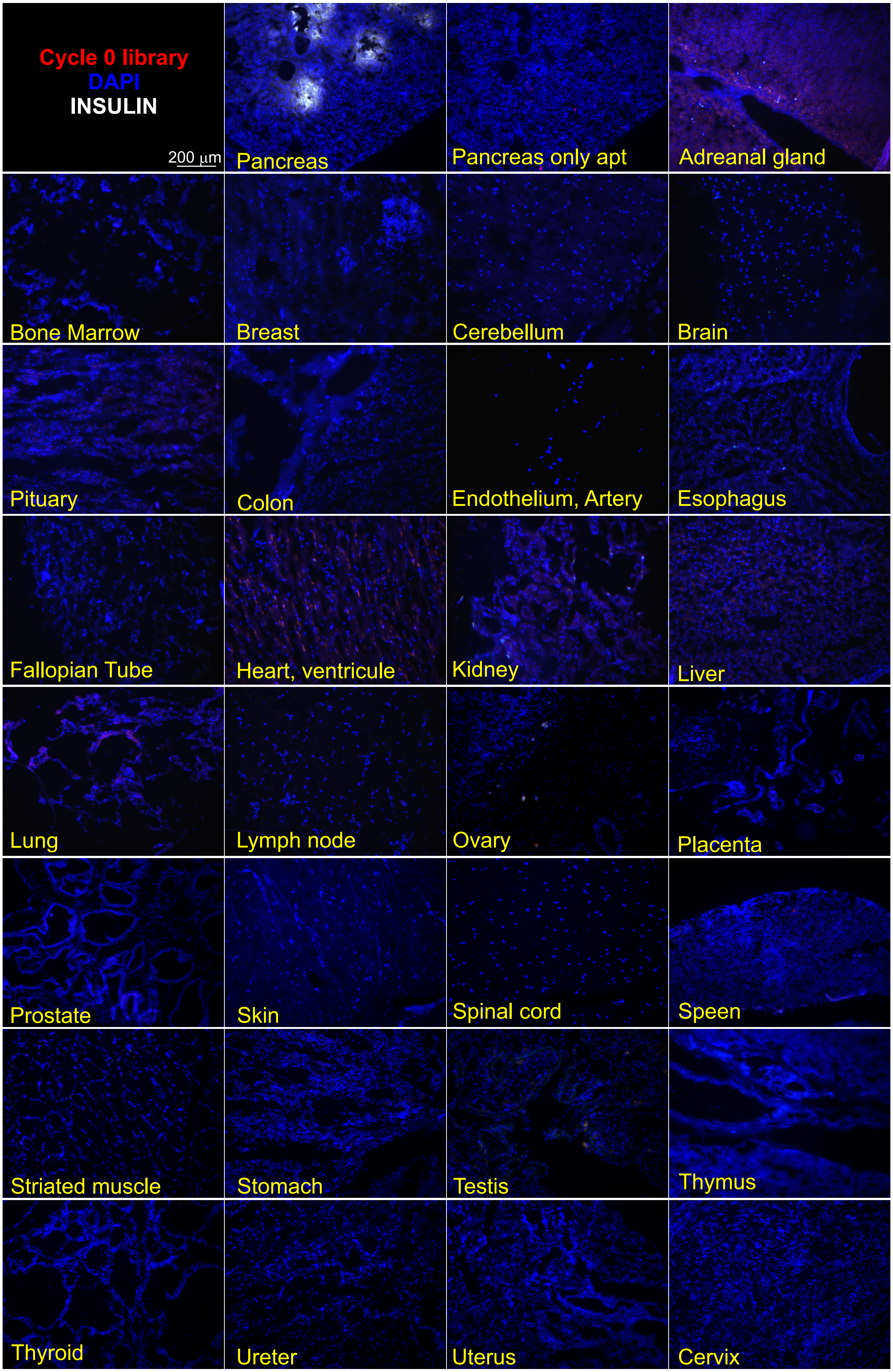

Supplementary figure 2 end

a

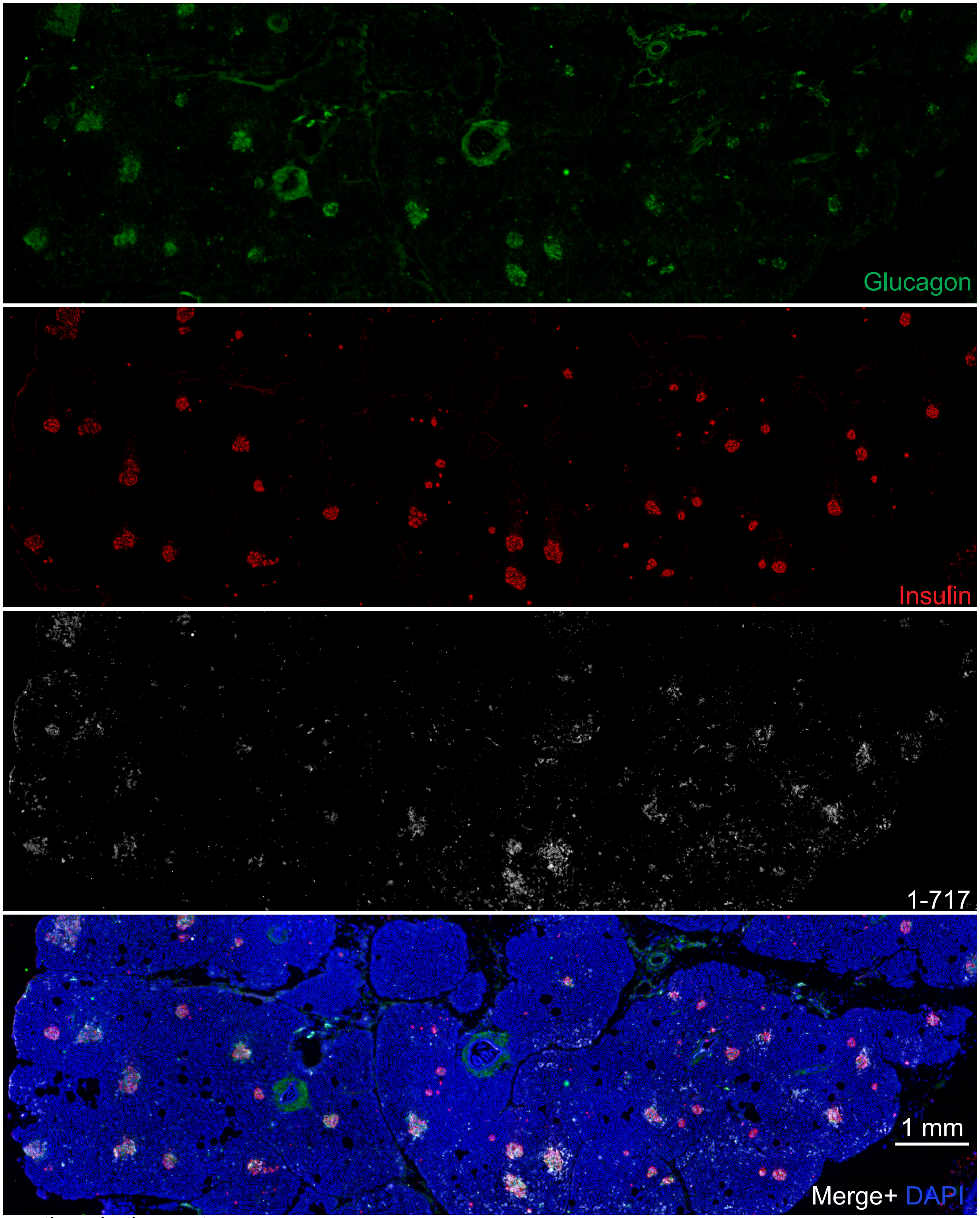

continue in the next pages

Supplementary figure 3: Scanned images of human pancreas sections stained with aptamer 1-717, m12-3773, or scrambled aptamers. Sections of human pancreases were stained with aptamer 1-717 (**a**), aptamer m12-3773 (**b**), or scrambled aptamers (**c**) and counter-stained with DAPI and antibodies against insulin and glucagon. Images were acquired with a high-definition scanner. **d**) confocal microscopy image of islets stained with cy3 labeled aptamer 1-717 or m12-3773. One representative image of two independent experiments is shown

b

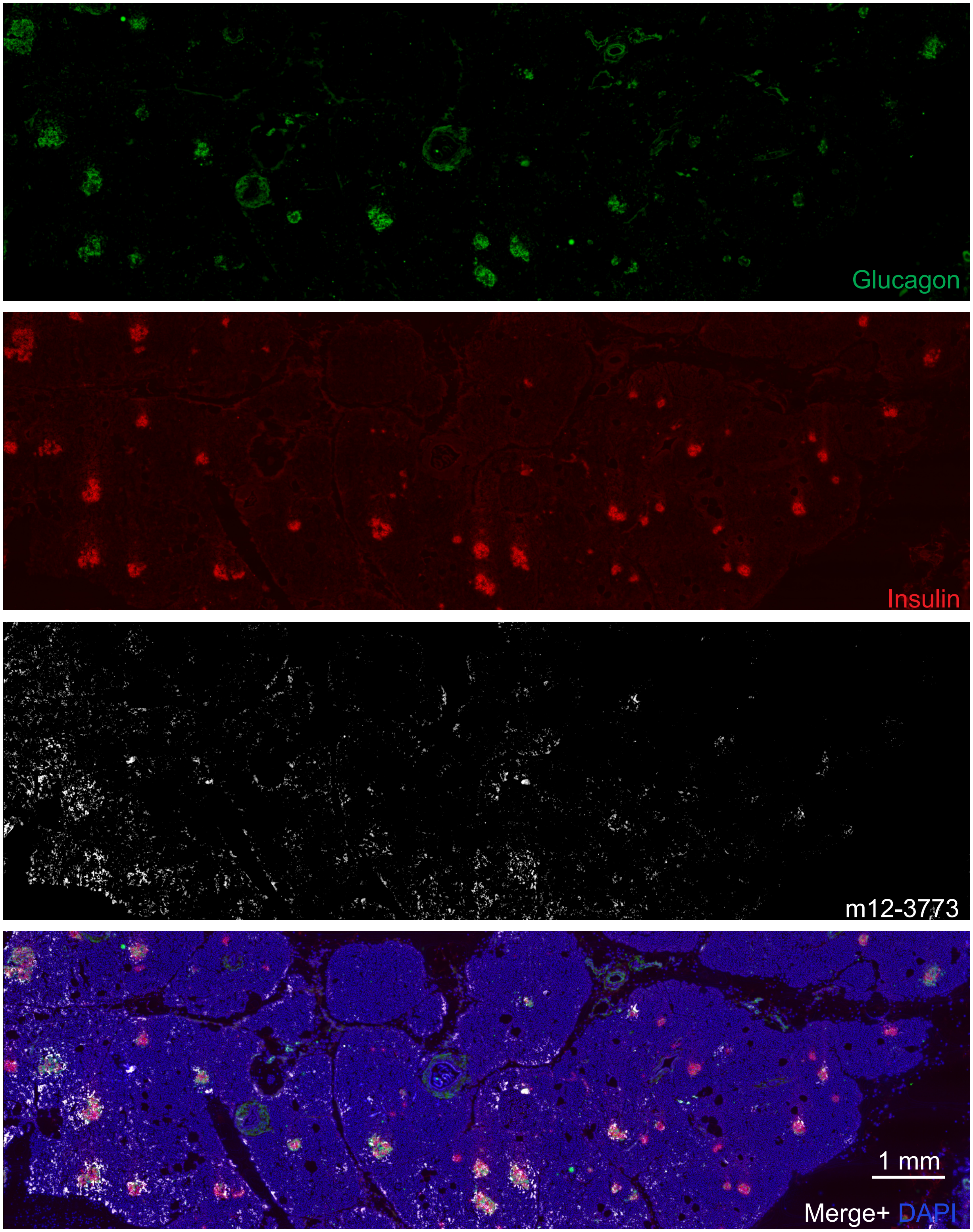

Supplementary figure 3b

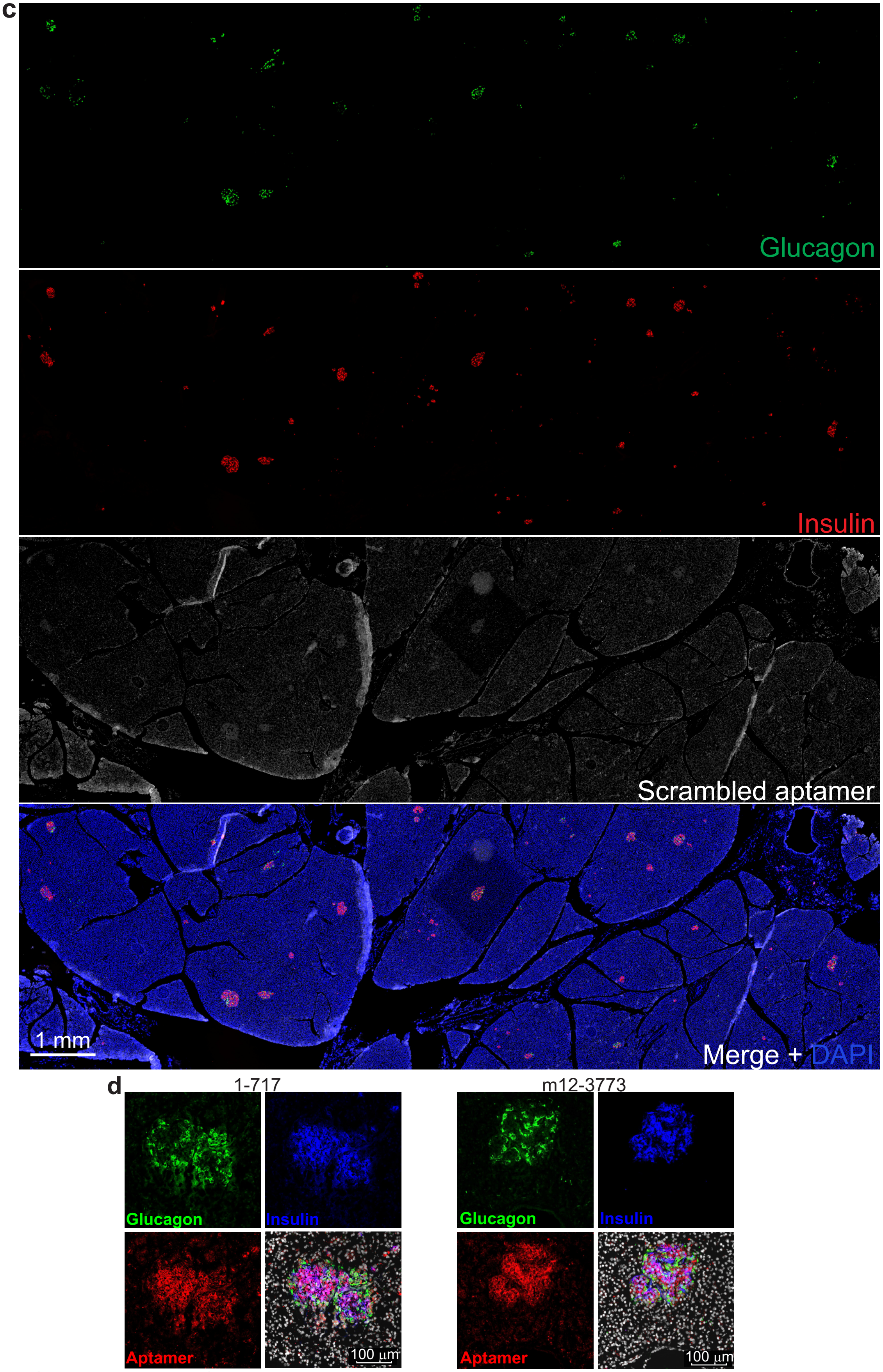

Supplementary figure 3 c and d

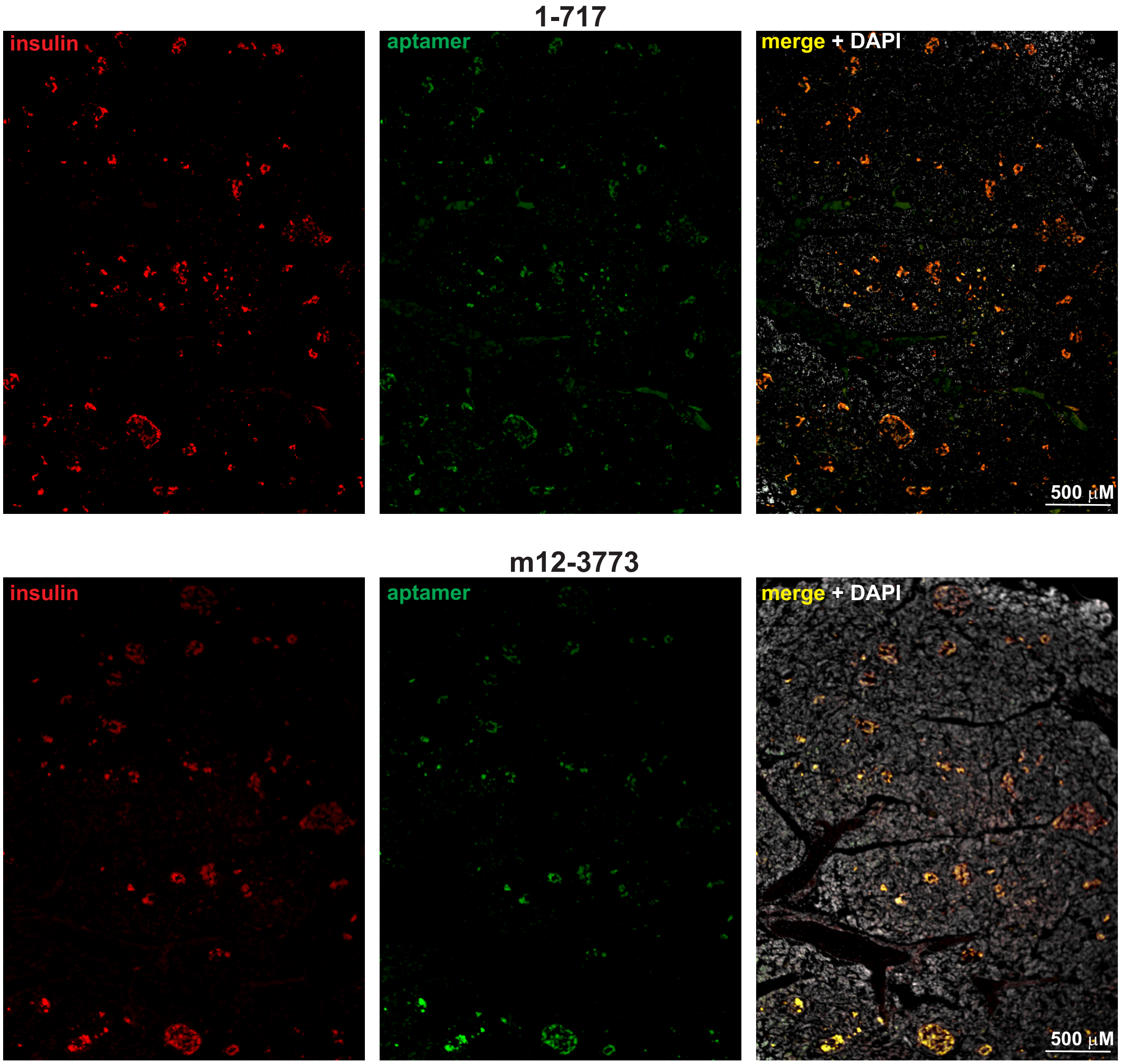

Supplementary figure 4. An optimized immune fluorescence staining technique shows that aptamer 1-717 and m12-3773 are highly specific for human islets. Snap frozen human pancreatic tissues were fixed with formalin and incubated with superblock-BSA-tween20 and dextran sulfate to reduce non-specific binding. Autofluorescence was quenched with image-it and endogenous biotin blocked with commercial kits. Then, tissues were stained with biotinylated aptamer, washed, and counterstained with anti-insulin antibodies and DAPI. Images were acquired at 4X in Keyence fluorescence microscopy. One representative image of two independent experiments is shown.

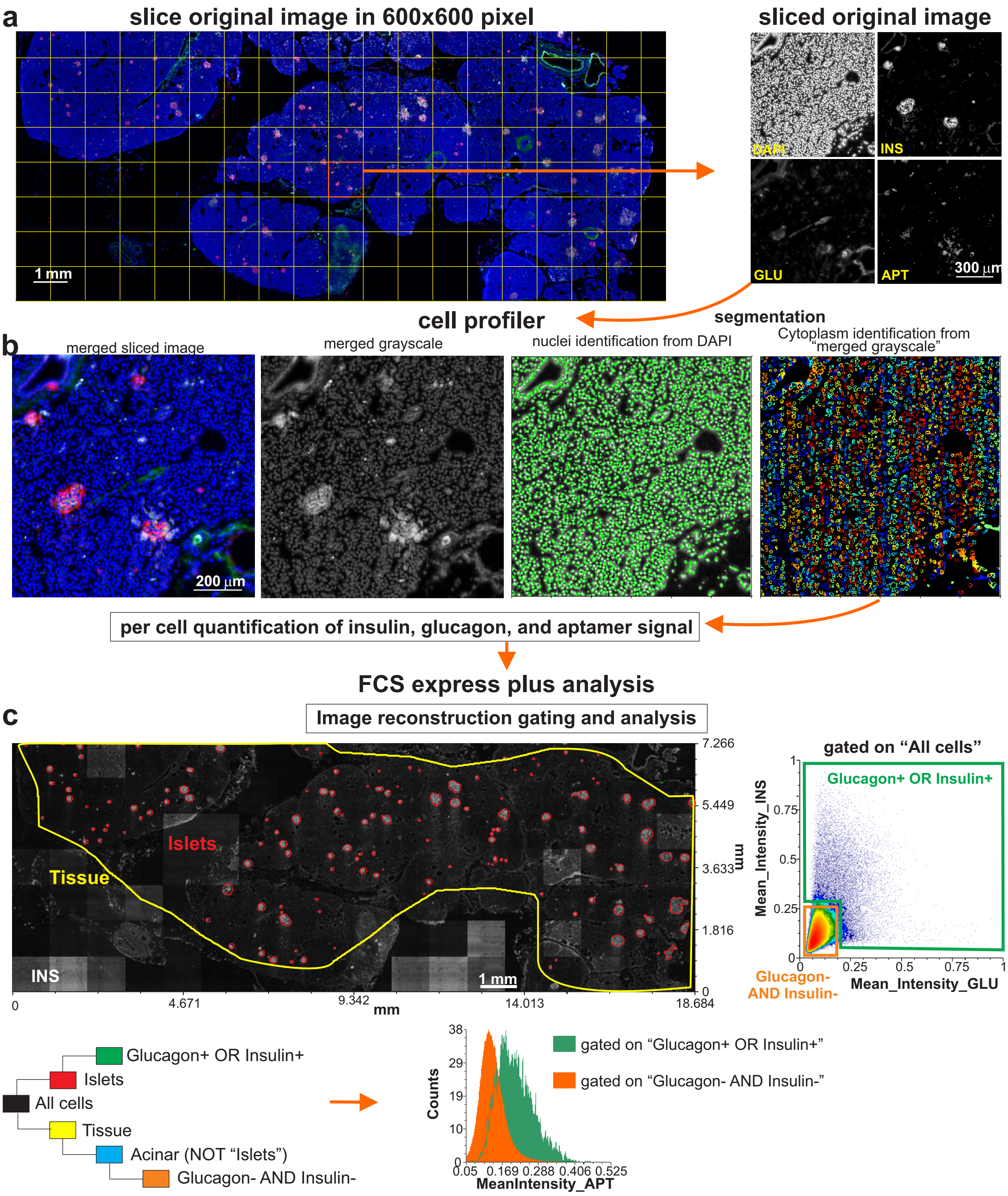

Supplementary figure 5: Example of image cytometry analysis. Section of human pancreas stained with DAPI, Cy3-labeled aptamer 1-717, and anti-insulin and anti-glucagon antibody were scanned with a fluorescence scanner. Images were optimized using CLAHE in ImageJ, each channel converted in grayscale, and sliced using image slicer (**a**). Images were segmented and analyzed in cellprofiler (**b**) and fed into FCSexpress v.7 for final analysis (**c**). The gating strategy is shown. One representative image from one of two independent experiments is shown in each panel.

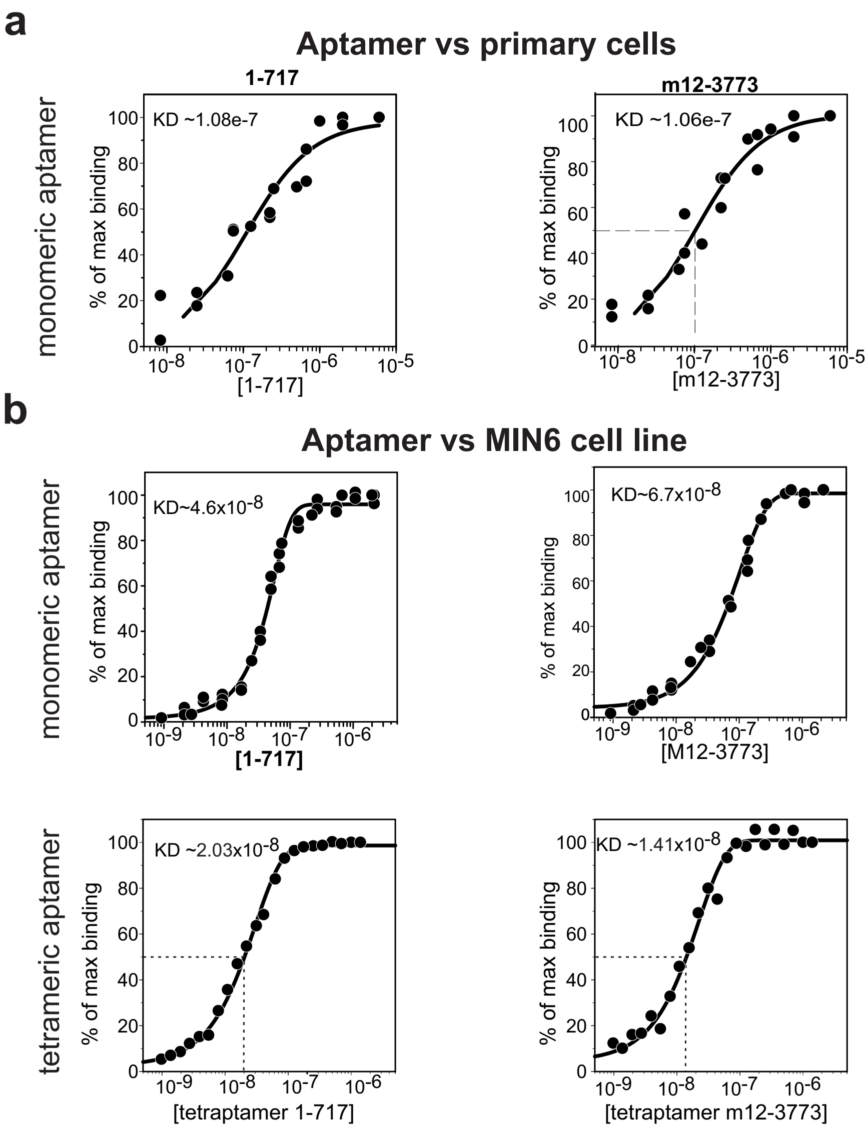

Supplementary figure 6: Affinity of aptamer m12-3773 and 1-717 for human β cells and MIN6 cells. a) Single-cell suspensions of human islets were stained with different quantities of Cy3-labeled aptamers, counterstained with vital dye and antibodies against insulin and glucagon, and analyzed by flow cytometry. **b)** MIN6 cells were stained with vital dye and either aptamer chimera annealed to cy5-labeled guide saRNA or biotinylated aptamers conjugated to AF647-streptavidin (tetrameric aptamer). Binding was analyzed by flow cytometry and data normalized on maximal MFI. Data derived from 2 independent experiments.

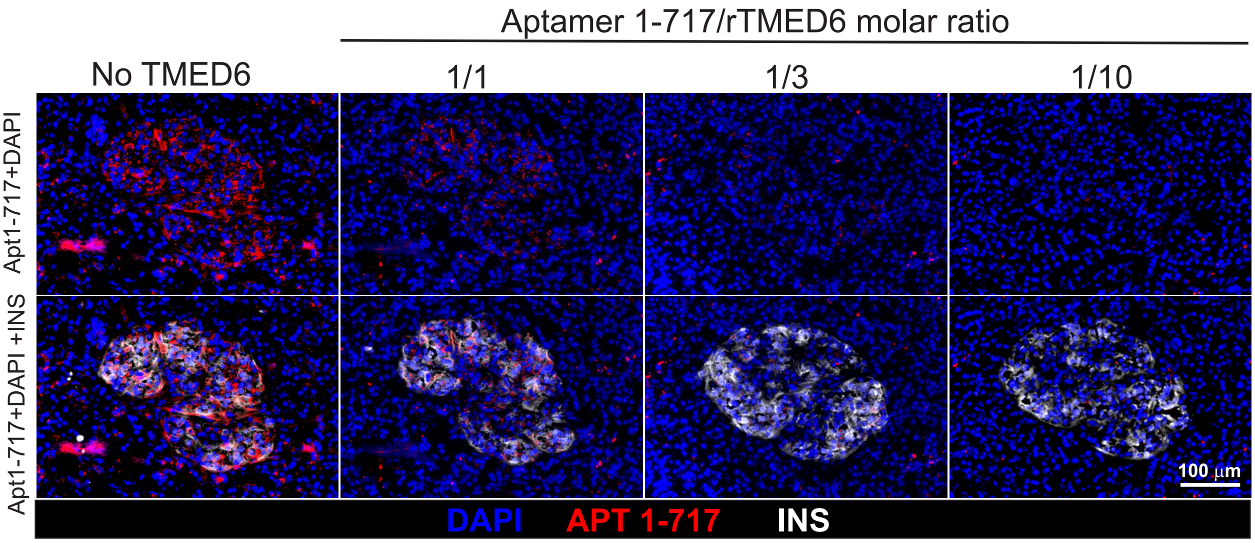

Supplementary figure 7: Cold target inhibition assays indicate TMED6 as a putative target for aptamer 1-717. Slides from human pancreatic tissue were stained with Cy3-labeled aptamer 1-717 in the presence of increasing molar ratios of recombinant TMED6 protein and counterstained anti-insulin antibody and DAPI. Images were acquired and processed with the identical setting. One representative image of two independent experiments is shown.

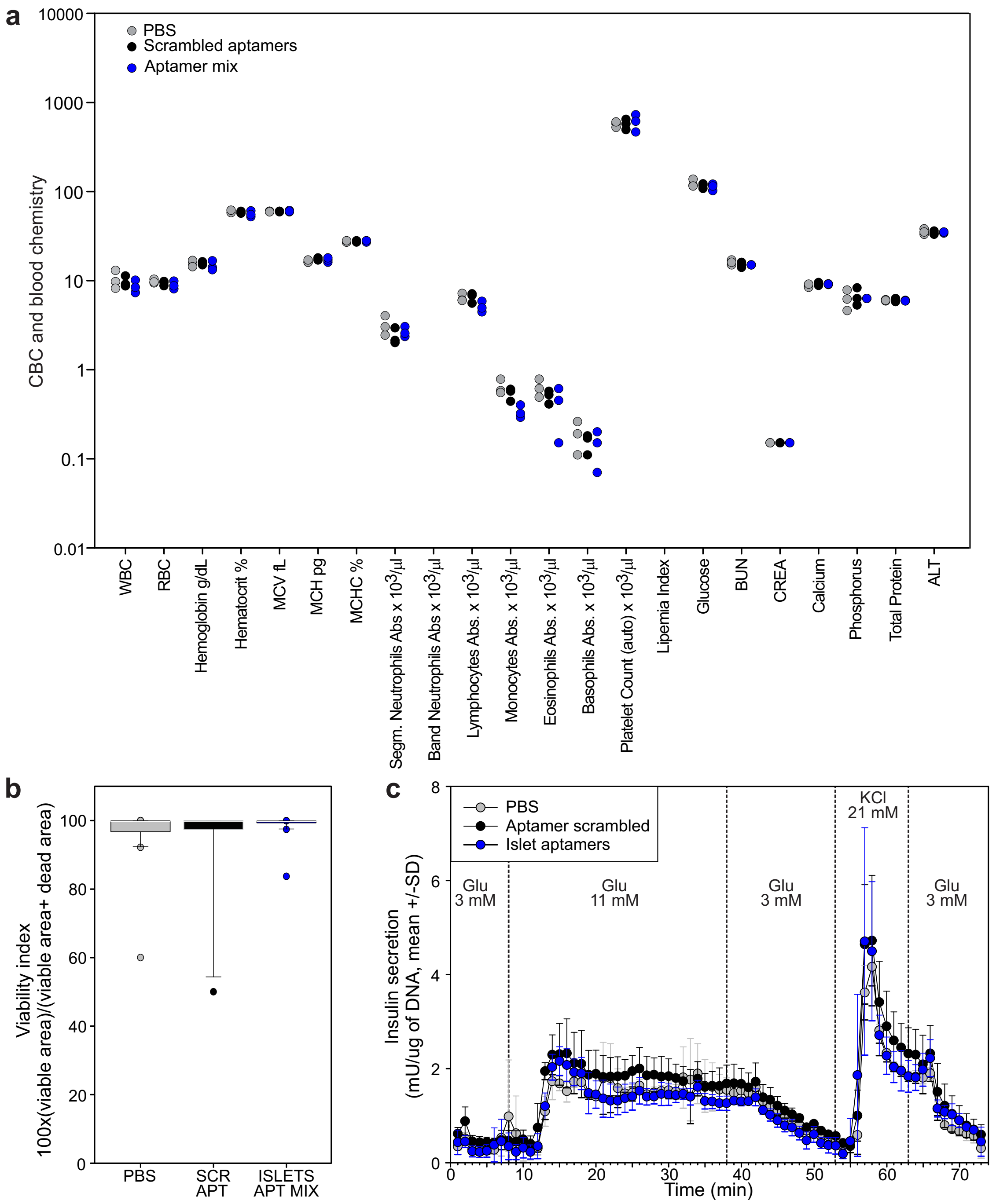

Supplementary figure 8: Aptamer 1-717 and m12-3773 do not show toxicity in vivo or in vitro. a) *Aptamer administration does not show toxicity in vivo.* Balb/c mice were injected daily with a mixture of aptamer m12-3773 and 1-717 (25 pMoles/g) for five days. Two days later, mice were euthanized toxicity evaluated in the blood from the indicated parameters. **b)** *Aptamers do not affect the viability of human islets.* Human islets 250 IEQ were incubated with one ml of PIM(r) media containing aptamer m12-3773 and 1-717 (2.5 nMoles) or scrambled aptamers for 24 hours. Islets were washed, and aliquots were evaluated for viability using the Fluorescein Diacetate/ Propidium Iodide (FDA)/(PI) Viability Assay. Data derived from 20, 10, and 21 islets treated with PBS, scrambled aptamers, or a mixture of aptamer m12-3773 and 1-717, respectively. The box plot shows the median, the 25th, 75 th, 10 th, 90 th percentile, and each outlier. **c)** *Aptamers do not affect insulin secretion from human islets.* Perifusion experiments were performed on islets from b as described in the material and methods. Mean, and standard deviation from n=3 biological independent samples are shown.

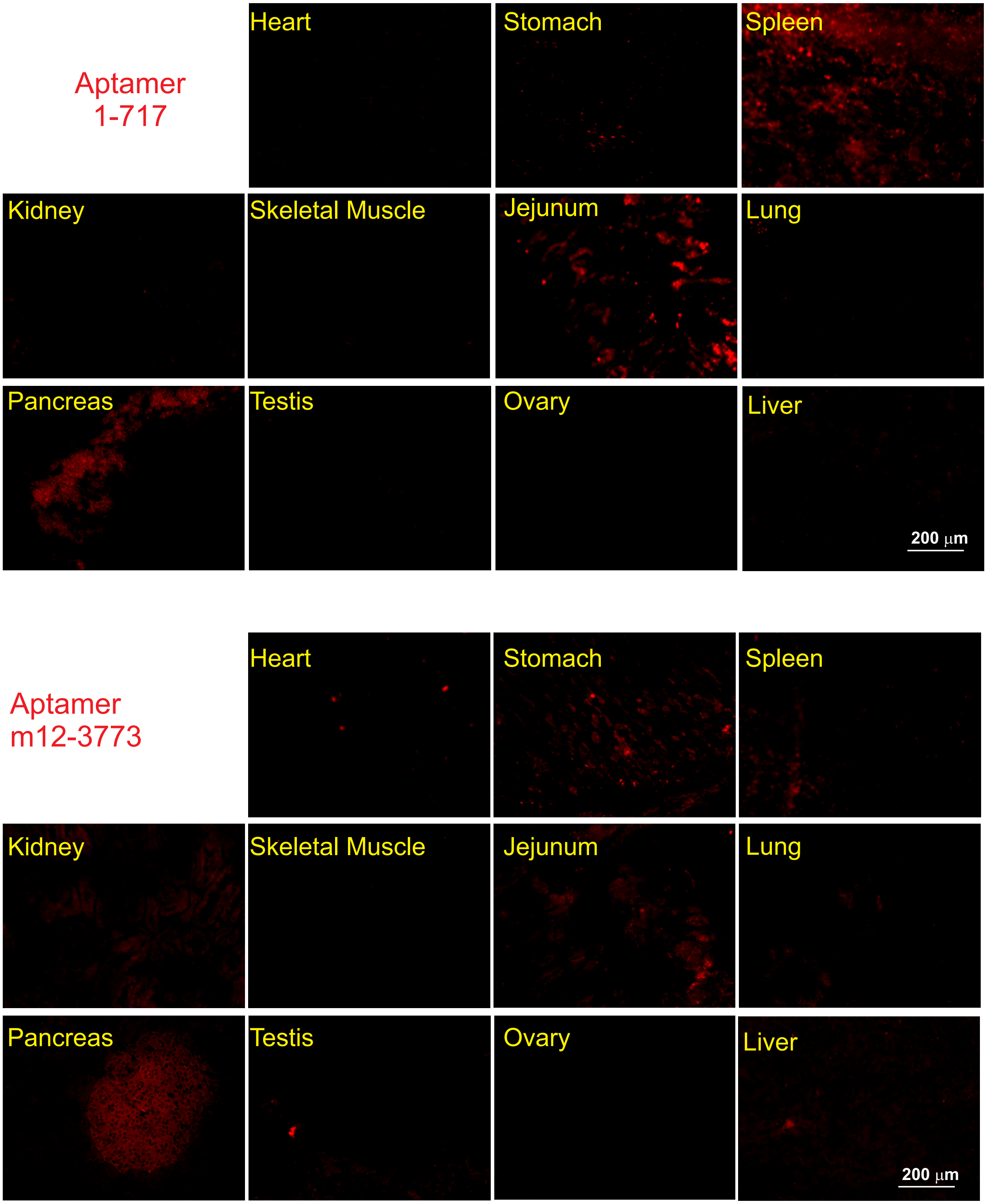

Supplementary figure 9: Aptamers 1-717 and m12-3773 cross-react with mouse tissues. Snap frozen mouse tissues arrays were stained with the indicated Cy3 labeled aptamers. Images were acquired with a Zeiss Apotome fluorescence microscope. One representative image of two independent experiments is shown

Supplementary table 1: Aptamer selected from the "Human cluster cell SELEX."

		Frequency								
aptamer	variable region sequence	cycle 0	cycle 1	cycle 2	cycle 3	cycle 4	cycle 5	cycle 6	cycle 7	cycle 8
1-717	UAAUUCUCAGGAGGUGCGGAACGGGAUAUGGAUUGUUCGC	BDL	4.49E-04	3.90E-04	8.23E-04	7.47E-04	2.40E-03	4.08E-03	8.20E-04	6.42E-04
6-2192	CAACAAACUAAUCAGACACGAGACAGAGAGAUAGAUCUGC	BDL	8.61E-04	1.05E-03	2.26E-03	1.05E-03	2.62E-03	1.68E-03	6.45E-04	5.43E-04
3-1183	CUUCCCUAUUCCAAAGGAGGUGCGGUACGUUUUGUUACGC	BDL	2.17E-04	1.71E-04	3.93E-04	2.05E-04	2.36E-04	1.82E-04	1.39E-04	1.47E-04
12-321	UGUACACUGAUUGCCUUUGUGUUAUGAGCGACAGAUCUGC	6.27E-08	3.44E-03	7.45E-03	9.49E-03	5.10E-03	1.01E-02	8.85E-03	2.84E-03	2.73E-03
15-1511	UUAUGCGUUUAAGUCAUUGACGCGUUACACUGGAGGGGGC	BDL	9.02E-04	1.70E-03	2.26E-03	1.28E-03	3.27E-03	2.76E-03	9.32E-04	9.15E-04
59-571	CCCAUCACUCCCGCGUAUUGCGAACGCAUCGUUAUUAGCCGU	BDL	1.11E-05	7.83E-05	2.28E-04	3.67E-04	4.85E-04	7.76E-04	1.49E-04	5.59E-05
64-2437	CCCAUCACUCCCGCGUAUUGCGAACGCAUCGUUAUUUAGCCGU	BDL	1.24E-03	9.44E-03	2.81E-02	4.45E-02	6.35E-02	1.02E-01	1.88E-02	7.42E-03
65-1192	CAGGUGCGGGAUCUAAUGCGUAGACAGCCAUAUACUGACA	BDL	2.18E-03	2.19E-03	2.30E-03	7.64E-04	7.45E-04	3.64E-04	2.35E-04	1.93E-04
68-1479	GCUGUGCCGGCCCUGCUCUGGUCGCCAUUGUCAGUCUGUG	BDL	2.21E-04	3.70E-05	2.22E-04	4.24E-05	1.23E-05	9.97E-05	6.98E-05	9.62E-05
71-2381	AUUACCAACUUGAACGCCGAGAGUGUGGUCACGUGUUCUG	BDL	2.31E-05	3.36E-05	3.44E-05	2.80E-05	7.24E-05	7.52E-05	2.82E-05	2.13E-05
86-323	ACGGAGGAUAGUUGCUAAUCGAGCCCUGCCGACGCUU	BDL	7.65E-04	9.87E-04	2.05E-03	9.69E-04	1.62E-03	9.65E-04	4.18E-04	4.27E-04
98-550	ACGGUUUCACCUCUAGGAGCACUGAAAGCCAACCUUCGCGCA	BDL	4.23E-05	9.20E-05	1.19E-04	6.61E-05	2.72E-04	3.58E-04	1.32E-04	8.91E-05
106-317	GGAAGCAACACUUAGUCGGGAUUGAUACGUGCCCAGUCAG	BDL	1.22E-04	3.03E-04	5.68E-04	4.33E-04	7.92E-04	5.95E-04	9.76E-05	9.34E-05
107-901	GGAAGCAACACUUAGUCGCGAUUGAUACGUGCGCAGUCAU	9.92E-07	5.58E-03	5.06E-03	9.89E-03	6.76E-03	8.06E-03	7.10E-03	1.72E-03	2.16E-03
109-2031	UGAAUUCUUCCGGCACUUUGUCAUCUUCACCCCCAUGCUGCA	BDL	2.85E-04	1.87E-03	3.57E-03	4.18E-03	1.68E-02	2.07E-02	1.99E-02	1.51E-02
128-3131	CAAAAACUGAUAAACACAGGUCCGGCAUUUGAGCGUACACC	BDL	2.16E-04	2.22E-04	4.71E-04	1.74E-04	4.81E-04	3.50E-04	9.65E-05	1.62E-04
133-1487	CCAGCCACACUUUGACCGAAUUGGCAAGCGCGGGCAAAUCGAA	BDL	3.05E-04	2.92E-04	7.80E-04	3.31E-04	2.82E-04	1.27E-04	9.01E-05	1.29E-04
141-47	CCGAUUUCGUCAUCCUCCAUACCAUCGCCUCCGUUCCGCGU	BDL	2.33E-07	1.74E-06	8.41E-06	2.58E-05	2.48E-05	3.25E-05	3.92E-05	5.39E-05
142-106	UACACUCAGUCACGUAGCACCGCAGUGACCCUUUGUACCG	BDL	5.13E-07	3.12E-07	4.99E-07	8.88E-08	7.41E-07	1.57E-06	1.22E-04	1.31E-04
144-445	CCGAUUUCGUCAUCCUCCAUACCAUCGCCUACCGUUCCGCGU	BDL	1.29E-05	1.40E-04	7.82E-04	1.82E-03	1.89E-03	2.28E-03	2.34E-03	2.45E-03
147-299	CCGAUUUCGUCAUCCUCCAUCAUCGCCUUACCGUUCCGCGU	BDL	7.46E-07	6.51E-06	3.14E-05	6.64E-05	6.72E-05	6.76E-05	8.57E-05	7.99E-05
154-16	CCGAUUCGUCAUCCUCCAUACCAUCGCCUUACCGUUCCGCGU	BDL	2.66E-06	3.06E-05	1.58E-04	3.55E-04	3.06E-04	3.49E-04	3.59E-04	3.79E-04
155-1103	CCGAUUUUCGUCAUCCUCCAUACCAUCGCCUUACCGUUCCGCGU	BDL	8.49E-06	1.05E-04	5.05E-04	1.06E-03	1.13E-03	1.22E-03	1.36E-03	1.17E-03
160-405	CCGAUUUCGUCAUCCUCCAUACCAUCGCCUUACCGUUUCCGCGU	BDL	5.13E-07	6.01E-06	3.17E-05	7.13E-05	7.72E-05	8.06E-05	9.27E-05	8.73E-05
162-674	CCGAUUUCGUCAUCCUCCAUACAUCGCCUUACCGUUCCGCGU	BDL	1.96E-06	1.93E-05	9.10E-05	2.21E-04	2.32E-04	2.93E-04	2.89E-04	3.06E-04
164-156	CCGAUUUCGUCAUCCUCCUACCAUCGCCUUACCGUUCCGCGU	BDL	2.98E-06	3.28E-05	1.71E-04	4.06E-04	4.29E-04	5.35E-04	6.39E-04	7.64E-04
166-279	CCGAUUUCGUCAUCCUCCAUACCAUCGCCUUACCGUUCCGCGU	BDL	2.90E-03	2.98E-02	1.54E-01	3.76E-01	3.29E-01	3.62E-01	3.95E-01	4.24E-01
170-1856	AUUAUUGUUUGACGUAUUCCAAGUGAGAUUACGCACGCAC	BDL	9.50E-05	8.43E-05	1.70E-04	8.25E-05	2.34E-04	1.97E-04	6.58E-05	1.14E-04
173-2273	ACCUUGUUUUCCUCUGUACCCCACUUCCCCAUUUCUCCCUGCU	BDL	8.37E-04	6.73E-03	1.29E-02	8.49E-03	1.38E-02	1.13E-02	5.55E-03	2.78E-03
198-370	CCGUCUCGCUCUCAUCCCAUGCACGAAACCUCUCUCCCUGCA	BDL	1.40E-07	7.17E-07	1.21E-06	8.88E-07	1.21E-04	4.43E-04	1.57E-04	2.50E-04
208-2529	CCGUCUCGCUCUCAUCCCAUGCACGAAACCUCUCUCACUGCA	BDL	1.93E-04	1.28E-03	2.21E-03	1.97E-03	4.82E-03	7.25E-03	1.08E-02	1.66E-02

BDL; Below detection limit

Supplementary table 2: Aptamer selected from the “Toggle cell SELEX.”				
		Frequency		fold change
**aptamer**	**sequence of the variable region**	**Cycle M8**	**Cycle M8H2**	
m3623	CAUAACAAUCGUUCCUGUGUGCCAAUCGCGGUCACGUCGCA	BDL	1.24E-07	NA
m4275	UUAUGCGUUUAAGUCAUUGACGCGUUACACUGGAGGGGGC	6.39E-05	1.54E-03	24.10
m4097	CAAAAACUGAUAAACACAGGUCCGGCAUUUGAGCGUACACC	1.27E-05	2.24E-04	17.64
m3845	ACGGAAGGAUAGUUGCUAAUCGAGCCCUGCCGACGCUU	1.69E-05	2.05E-04	12.13
m2548	CGCAUCACUCCCGCGUAUUGCGAACGCAUCGUUAUUUAGCCGU	1.63E-05	1.91E-04	11.72
m3265	ACGGAGGAUAGUUGCUAAUCGAGCCCUGCUGACGCUU	9.38E-06	1.11E-04	11.83
m2929	CAACAAACUAAUCAGACACGAGGCAGAAAGAUAGGUCCGG	7.52E-06	1.07E-04	14.23
m3565	AUUAUUGUUUGACGUAUUCCAAGUGAGAUUACGCACGCACCAGA	7.04E-06	8.46E-05	12.02
m1723	GGAAGCAACACUUAGUCGGGAUUGAUACGUGCCCAGUCAGCAGA	5.44E-06	6.29E-05	11.56
m3997	UAAUUCUCAGGAGGUGCGGAACGGGAUAUGGAUUGUUCGCCAGU	4.62E-03	1.66E-02	3.59
m1253	UAAUUCUCAGGAGGUGCGGAACGGGAUAUGGAUUGUUCGCCAUA	3.26E-04	1.21E-03	3.71
m3111	CCGAUUUCGUCGUCCUCCAUACCAUCGCCUUACCGUUCCGCGU	3.95E-04	9.84E-04	2.49
m2554	CCCAUCACUCCCGAGUAUUGCGAACGCAUCGUUAUUUAGCCGU	3.44E-04	8.62E-04	2.51
m2117	CCCAUCACUCCCGCGUAUUGCGAACGCAUCGUUAUUUAGCAGU	3.27E-04	8.26E-04	2.53
m2340	CCGAUUUCAUCAUCCUCCAUACCAUCGCCUUACCGUUCCGCGU	3.36E-04	7.98E-04	2.38
m1874	CCGAUUUCGUCAUCCUCCAUACCAUCGCAUUACCGUUCCGCGU	2.53E-04	7.86E-04	3.11
m1803	UAAUUCUCAGGAGGUGCGGAACGGGAUAUGGACUGUUCGCCAGU	1.37E-04	5.18E-04	3.78
m3211	CCCAUCACUCACGCGUAUUGCGAACGCAUCGUUAUUUAGCCGU	1.23E-04	4.85E-04	3.94
m2119	ACCAUCACUCCCGCGUAUUGCGAACGCAUCGUUAUUUAGCCGU	1.45E-04	4.60E-04	3.17
m1874	CCGAUUUCGUCAUCCUCCAUACCAUCGCCUUACAGUUCCGCGU	1.94E-04	4.18E-04	2.15
m2717	UAAUUCUCAGGAGGUGCGGAACGGGAUAUGGACUGUUCGCCAGA	1.81E-04	3.87E-04	2.14
m2753	CCGAUUUCGUCAUACUCCAUACCAUCGCCUUACCGUUCCGCGU	1.31E-04	3.47E-04	2.65
m3500	CCCAUCACUCCCGCGUAUAGCGAACGCAUCGUUAUUUAGCCGU	1.21E-04	3.12E-04	2.58
m2131	CCCAUCACUCCGGCGUAUUGCGAACGCAUCGUUAUUUAGCCGU	1.31E-04	2.91E-04	2.22
m552	CCCAACACUCCCGCGUAUUGCGAACGCAUCGUUAUUUAGCCGU	1.23E-04	2.58E-04	2.10
m1861	CCGAUUUCGUCAUCCUCCAUACAUCGCCUUACCGUUCCGCGU	1.13E-04	2.38E-04	2.11
m1615	CCCAUCACUCCCGCGUAUUGCGAUCGCAUCGUUAUUUAGCCGU	1.08E-04	2.36E-04	2.19
m12	CCGAUUUCGUCAUCCUCCAUACGAUCGCCUUACCGUUCCGCGU	1.08E-04	2.30E-04	2.13
m1-2623	UACACUCAGUCACGUAGCACCGCAGUGACCCUUUGUACCG	BDL	2.97E-04	NA
m3-2228	UAAUUCUCAGGAGGUGCGGAACGGGAUAUGGGUUGUUCGCCAGU	7.82E-05	3.78E-04	4.83
m4-2502	CCCAUCACUCCCGCGUAUUGCGAACGCAUCGUUAUUUAGCCAGA	2.92E-07	2.76E-04	945.21
m4-3211	CCCAUCACUCGCGCGUAUUGCGAACGCAUCGUUAUUUAGCCGU	4.09E-05	2.50E-04	6.11
m5-3229	CCUAGUACAAAAGCCUGAUCUCUGUGAGCAGACACUAGAACAGA	BDL	2.20E-04	NA
m7-2539	AUUACCAACUUGAACGCCGAGAGUGUGGUCACGUGUUCUGCAGA	BDL	1.94E-03	NA
m9-3076	GGAAGCAACACUUAGUCGCGAUUGAUACGUGCGCAGUCAUCAGA	BDL	5.84E-03	NA
m10-3248	UGUACACUGAUUGCCUUUGUGUUAUGAGCGACAGAUCUGCCAGA	BDL	1.82E-03	NA
m12-3773	CAACAAACUAAUCAGACACGAGACAGAGAGAUAGAUCUGCCAGA	BDL	2.08E-03	NA
m24-3219	CAGGUGCGGGAUCUAAUGCGUAGACAGCCAUAUACUGACACAGA	BDL	7.54E-04	NA
m30-3568	AACAGCUUAAUCGCCAGUCGAUACGCGCCAUACAUCAUCACAGA	BDL	1.05E-03	NA

BDL= below detection limit
NA= not available

Supplementary table 3: primers and aptamer template sequences	
**Sequence id**	**Sequence**
Sullenger library	TCTCGGATCCTCAGCGAGTCGTCTGNNNNNNNNNNNNNNNNNNNNNNNNNNNNNNNNNNNNNN NNCCGCATCGTCCTCCCTA
SUL3'	TCTCGGATCCTCAGCGAGTCGTC
SUL3'short	TCTCGGATCCTCAGCGAGTCG
SUL5'	GGGGGAATTCTAATACGACTCACTATAGGGAGGACGATGCGG
TMED6 SUL3'	GGAGCTGATCGATATGACTGGGTCTCGGATCCTCAGCGAGTCGTC
CLU SUL3'	AGGAAGAACCCTAAATTTAGGGTCTCGGATCCTCAGCGAGTCGTC
Control SUL3''	TAAGGCTATGAAGAGATACGGGTCTCGGATCCTCAGCGAGTCGTC
TMED6 guide	rGrGrArGrCrUrGrArUrCrGrArUrArUrGrArCrUrUrU
CLU Guide	rArGrGrArArGrArArCrCrCrUrArArArUrUrUrArUrU
Control Guide	rTrArArGrGrCrTrArTrGrArArGrArGrArTrArCrUrU
ILL-PF1 A	ACACTCTTTCCCTACACGACGCTCTTCCGATCTCTAGTGAGGACGATGCGG
ILL-PF2 A	ACACTCTTTCCCTACACGACGCTCTTCCGATCTAGCTGGAGGACGATGCGG
ILL-PF3 A	ACACTCTTTCCCTACACGACGCTCTTCCGATCTTCGACGAGGACGATGCGG
ILL-PF4 A	ACACTCTTTCCCTACACGACGCTCTTCCGATCTGATCAGAGGACGATGCGG
ILL-PFU B	AATGATACGGCGACCACCGAGATCTACACTCTTTCCCTACACGACGCTCTTCCGATCT
ILL-PRU A	ATCTCGTATGCCGTCTTCTGCTTGTCTCGGATCCTCAGCGAGTC
ILL-PR2 B	GATCGGAAGAGCACACGTCTGAACTCCAGTCACCGATGTATCTCGTATGCCGTCTTCTGCTTG
ILL-PR1 B	GATCGGAAGAGCACACGTCTGAACTCCAGTCACATCACGATCTCGTATGCCGTCTTCTGCTTG
ILL-PR3 B	GATCGGAAGAGCACACGTCTGAACTCCAGTCACTGACCAATCTCGTATGCCGTCTTCTGCT
ILL-PR4 B	GATCGGAAGAGCACACGTCTGAACTCCAGTCACGCCAATATCTCGTATGCCGTCTTCTGCTTG
166-279 template	TCTCGGATCCTCAGCGAGTCGTCTGACGCGGAACGGTAAGGCGATGGTATGGAGGATGACGAA ATCGGCCGCATCGTCCTCCCTA
109-2031 template	TCTCGGATCCTCAGCGAGTCGTCTGTGCAGCATGGGGGTGAAGATGACAAAGTGCCGGAAGAA TTCACCGCATCGTCCTCCCTA
208-2529 template	TCTCGGATCCTCAGCGAGTCGTCTGTGCAGTGAGAGAGGTTTCGTGCATGGGATGAGAGCGAG ACGGCCGCATCGTCCTCCCTA
64-2437 template	TCTCGGATCCTCAGCGAGTCGTCTGACGGCTAAATAACGATGCGTTCGCAATACGCGGGAGTG ATGGGCCGCATCGTCCTCCCTA
173-2273 template	TCTCGGATCCTCAGCGAGTCGTCTGAGCAGGGAGAAATGGGGAAGTGGGGTACAGAGGAAAAC AAGGTCCGCATCGTCCTCCCTA
12-2617 template	TCTCGGATCCTCAGCGAGTCGTCTGGCAGATCTGTCGCTCATAACACAAAGGCAATCAGTGTA CACCGCATCGTCCTCCCTA
107-901 template	TCTCGGATCCTCAGCGAGTCGTCTGATGACTGCGCACGTATCAATCGCGACTAAGTGTTGCTT CCCCGCATCGTCCTCCCTA
155-1103 template	TCTCGGATCCTCAGCGAGTCGTCTGGGAACGGTAAGGCGATGGTATGGAGGATGACGAAAATC GGCCGCATCGTCCTCCCTA
1-717 template	TCTCGGATCCTCAGCGAGTCGTCTGGCGAACAATCCATATCCCGTTCCGCACCTCCTGAGAAT TACCGCATCGTCCTCCCTA
m1-2623 template	TCTCGGATCCTCAGCGAGTCGTCTGCGGTACAAAGGGTCACTGCGGTGCTACGTGACTGAGTG TACCGCATCGTCCTCCCTA
m5-3229 template	TCTCGGATCCTCAGCGAGTCGTCTGTTCTAGTGTCTGCTCACAGAGATCAGGCTTTTGTACTA GGCCGCATCGTCCTCCCTA
m7-2539 template	TCTCGGATCCTCAGCGAGTCGTCTGCAGAACACGTGACCACACTCTCGGCGTTCAAGTTGGTA ATCCGCATCGTCCTCCCTA
m9-3076 template	TCTCGGATCCTCAGCGAGTCGTCTGATGACTGCGCACGTATCAATCGCGACTAAGTGTTGCTT CCCCGCATCGTCCTCCCTA
m12-3773 template	TCTCGGATCCTCAGCGAGTCGTCTGGCAGATCTATCTCTCTGTCTCGTGTCTGATTAGTTTGT TGCCGCATCGTCCTCCCTA
m24-3229 template	TCTCGGATCCTCAGCGAGTCGTCTGTGTCAGTATATGGCTGTCTACGCATTAGATCCCGCACC TGCCGCATCGTCCTCCCTA
Scr-m12-3773 template	GGAGGACGATGCGGACCAACCACTGGATAACCAAGAGAGTACAAGAGATCACAGATGACGACT CGCTGAGGATCCGAGA
Scr-1-717 template	GGAGGACGATGCGGGGTAAGCATTCCTTATGATTGATAGTATGGAGCGGACGCGGCGACGACT CGCTGAGGATCCGAGA

Supplementary table 4: Donor and characteristics of islet preparation.

Donors (n=16)	25%ile	median	75%ile	range
**age**	40	49	57	23-63
**BMI**	20.8	27.5	33	18.2-33
**cold ischemia time (min)**	615	747	855	594-1152
**islet purity**	88.75	90	91.25	50-95
**islet viability**	90	90	90	88.75-91.25
**1-717 binding to β cell**<br>MFI (fold-over background)	2.49	3.31	4.47	1.57-12.96-
**m12-3773 binding to β cell**<br>MFI (fold-over background)	2.60	3.58	8.06	1.68-13.40

race	Black	White Non-Hispanic	White Hispanic
	31	25	44

gender	female	male
	50	50

Supplementary table 5: proteins isolated via aptamer mediated immune precipitation and mass spectrometry analysis

ID	Unipro#	MW	Cellular localization	Mascot Score			
				M12-3773 aptamer		Scrambled aptamer	
				Exp.1	Exp.2	Exp.1	Exp.2
**CLUS**	**P10909**	**52461**	CYTOPLASM/MEMBRANE/SECRETED	**265**	**302**	**ND**	**ND**
LMNA	P02545	74095	NUCLEUS	627	ND	ND	ND
LMNB	Q03252	67647	NUCLEUS	322	ND	ND	ND
ODP2	P10515	68953	MYTHOCONDRIA	179	ND	ND	ND
MyH9	P35579	226329	CYTOSKELETON/EXOSOME/SECRETED	67	ND	ND	ND
H3.2	Q71DI3	15379	NUCLEUS	ND	252	ND	ND
K22E	P35908	65393	CYTOPLASM/SECRETED	ND	187	ND	ND
K2C72	Q14CN4	55842	CYTOPLASM/SECRETED	ND	66	ND	ND
H2B2F	Q50NW6	13912	NUCLEUS	ND	56	ND	ND
H1.X	Q92522	22474	NUCLEUS	ND	52	ND	ND
CKAP4	Q07065	65983	MEMBRANE/ER	50	ND	ND	ND
MYH14	Q7Z406	227863	CYTOSKELETON	65	ND	ND	ND
H1.2	P16403	21352	NUCLEUS	ND	319	ND	ND
H1.0	P07305	20850	NUCLEUS	ND	225	ND	ND
UBIQ	P62988	8560	NUCLEUS	ND	105	ND	ND
CENPV	Q7Z7K6	29927	NUCLEUS	ND	76	ND	ND
NMD3A	Q8TCU5	125385	MEMBRANE	ND	69	ND	ND
THOC4	Q86V81	26872	NUCLEUS	ND	69	ND	ND
H1.5	P16401	22566	NUCLEUS	ND	60	ND	ND
K1C9	P35527	62027	CYTOPLASM/MEMBRANE/SECRETED	69	491	ND	282
K2C1	P04264	65999	MEMBRANE	167	804	864	523
K1C10	P13645	58792	SECRETED	53	369	433	240
ACTBM	Q9BYX7	41989		ND	ND	ND	96
ALBU	P02768	69321		ND	ND	ND	119
ANXA2	P07355	38580		ND	ND	ND	247
APEX1	P27695	35532		ND	ND	ND	65
CBPA1	P15085	47111		ND	ND	ND	197
EF1D	P26692	31103		ND	ND	ND	62
G3P	P04406	36030		ND	ND	ND	159
HCDH	Q16836	34256		ND	ND	ND	97
HNRH3	P31942	36903		ND	ND	ND	55
K2C8	P05787	53671		ND	ND	ND	74
MDHM	P40926	35481		ND	ND	ND	157
RA1L3	P0C7M2	34202		ND	ND	ND	100
ROA2	P22626	37407		ND	ND	ND	372
SSRA	P43307	32215		ND	ND	ND	73
ANR31	Q8N7Z5	210685		ND	ND	50	ND
IF6	P56537	26582		ND	ND	51	ND
KT81L	A6NCN2	53375		ND	ND	52	ND
NEUL	Q9BYT8	80600		ND	ND	55	ND
CBX3	Q13185	20798		ND	ND	62	ND
BRCA2	P51587	383986		ND	ND	66	ND
H4	P62805	11360		ND	ND	66	ND
H2BFS	P57053	13936		ND	ND	261	ND
H2A2A	Q6FI13	14087		ND	ND	389	ND
H2A1	P0C0S8	14083		ND	ND	421	ND
H2A3	Q7L7L0	14113		ND	ND	428	ND

ND: Not detected

Supplementary table 6: Candidate saRNAs for human Xiap

Position from Xiap TSS	Sequence	Wang Score	Effect size (qRT-PCR)	Position from Xiap TSS	Sequence	Wang Score	Effect size (qRT-PCR)
-124	TGCCTTTCTTCCACTATTC	4.5	-4.93	-586	TCCCATTTTCCTGAAACAA	4.5	-4.87
-126	TGTGCCTTTCTTCCACTAT	4.5	-4.96	-588	GATCCCATTTTCCTGAAAC	5	2.58
-132	TGTCCTTGTGCCTTTCTTC	4.5	1.64	-597	ACCCTGTAAGATCCCATTT	4.5	1.49
-136	TTTCTGTCCTTGTGCCTTT	4.5	2.1	-607	TCACCACATTACCCTGTAA	5	12.02
-141	CCTCTTTTCTGTCCTTGTG	4.5	5.95	-608	CTCACCACATTACCCTGTA	5.5	3.76
-190	CACAGTACCTGATCTATAG	4.5	0.53	-615	CCATCATCTCACCACATTA	5	2.38
-195	CCTAACACAGTACCTGATC	5	-4.78	-669	GGGAGGATCATATCTTGTA	5	2.75
-196	GCCTAACACAGTACCTGAT	4.5	3.94	-671	AAGGGAGGATCATATCTTG	4.5	1.15
-231	GGGTTGTCAACATTACAAC	5	2.04	-672	GAAGGGAGGATCATATCTT	4.5	2.01
-233	CAGGGTTGTCAACATTACA	5	0.83	-674	GTGAAGGGAGGATCATATC	4.5	-0.05
-234	ACAGGGTTGTCAACATTAC	5	4.38	-676	TTGTGAAGGGAGGATCATA	5.5	9.24
-244	TGCTTATCTTACAGGGTTG	5	1.15	-677	GTTGTGAAGGGAGGATCAT	4.5	9.16
-255	CCCAAAGACAGTGCTTATC	4.5	2.73	-688	CAAGTTCCCTTGTTGTGAA	4.5	0.9
-256	CCCCAAAGACAGTGCTTAT	4.5	2.07	-690	TCCAAGTTCCCTTGTTGTG	4.5	0.95
-257	CCCCCAAAGACAGTGCTTA	5	3.37	-692	CCTCCAAGTTCCCTTGTTG	4.5	-4.96
-268	GGCTTATTAAACCCCCAAA	5	1.88	-744	GGGGAGAATAAACATGCAA	5	-4.43
-269	GGGCTTATTAAACCCCCAA	4.5	-4.77	-751	CACCCAAGGGGAGAATAAA	5	16.69
-291	GCCTCAGTTTCCTTTGTAA	5.5	-4.77	-752	GCACCCAAGGGGAGAATAA	5.5	1.67
-292	GGCCTCAGTTTCCTTTGTA	6	1.34	-791	CACAAGGAGTAGTTCTGAT	4.5	2.91
-294	TGGGCCTCAGTTTCCTTTG	5.5	1.3	-792	GCACAAGGAGTAGTTCTGA	4.5	1.53
-350	CGATTCTAACCTCAGTTAC	4.5	4.72	-793	GGCACAAGGAGTAGTTCTG	5	6.635
-351	CCGATTCTAACCTCAGTTA	4.5	4.2	-826	TTAGCGGAAGGCAGGAAAA	4.5	1
-361	GGAAACAGATCCGATTCTA	4.5	3.19	-846	GAGGGAGTAGTTTGAACAA	6	5.23
-369	CCCTTCTTGGAAACAGATC	4.5	4.11	-847	AGAGGGAGTAGTTTGAACA	5	0.69
-374	TCCTTCCCTTCTTGGAAAC	4.5	4.2	-848	CAGAGGGAGTAGTTTGAAC	5	1.98
-375	ATCCTTCCCTTCTTGGAAA	5	3.32	-849	GCAGAGGGAGTAGTTTGAA	5.5	-4.62
-383	TCCCTTTCATCCTTCCCTT	4.5	1.54	-851	AGGCAGAGGGAGTAGTTTG	4.5	2.59
-395	CCTTTCTTGTCATCCCTTT	4.5	3.47	-856	CACATAGGCAGAGGGAGTA	5.5	1.06
-396	CCCTTTCTTGTCATCCCTT	4.5	-4.88	-928	TGTGTACGGGCAGTCTTTT	4.5	0.39
-403	AGGGTCTCCCTTTCTTGTC	4.5	3.09	-929	TTGTGTACGGGCAGTCTTT	4.5	1.6
-439	CCTTCACCTCCCAATCTTA	4.5	2.09	-964	GAGATGAACTTCAGCTAAG	5	1.87
-442	TTCCCTTCACCTCCCAATC	5.5	1.98	-966	GAGAGATGAACTTCAGCTA	5.5	15.03
-443	TTTCCCTTCACCTCCCAAT	5	2.4	-974	AGAGCATGGAGAGATGAAC	4.5	-6
-474	GGTCCATCTGCTTTCTTTT	4.5	1.21	-975	CAGAGCATGGAGAGATGAA	4.5	0.48
-478	TTCCGGTCCATCTGCTTTC	5	-4.93	-980	GAGTTCAGAGCATGGAGAG	4.5	-4.35
-498	TGTACACAGACTTCCTTTG	4.5	2.18	-994	GAGAAGTGCCACTAGAGTT	4.5	2.32
-517	GGGTATGACCAGCATTTTA	5.5	3.4	-1078	CCCAGCCAAAACAGTAAAT	5	1.36
-518	AGGGTATGACCAGCATTTT	5	3.17	-1079	GCCCAGCCAAAACAGTAAA	6	0.79
-519	CAGGGTATGACCAGCATTT	4.5	1.15	-1080	TGCCCAGCCAAAACAGTAA	6	2.11
-520	CCAGGGTATGACCAGCATT	5.5	2.47	-1081	ATGCCCAGCCAAAACAGTA	6	5.09
-521	TCCAGGGTATGACCAGCAT	5	1.83	-1084	ACCATGCCCAGCCAAAACA	4.5	8.67
-547	CAATCCTACAACGCATTTG	5	1.45	-1117	CCTCCCAAAGTGTTAGGAT	4.5	8.78
-550	TGACAATCCTACAACGCAT	4.5	4.77	-1118	GCCTCCCAAAGTGTTAGGA	4.5	3.54
-585	CCCATTTTCCTGAAACAAA	4.5	3.54	-1121	TCGGCCTCCCAAAGTGTTA	5	1.74

Note: saRNA giving an effect size >10 are highlighted in yellow
