# Supplementary material for: RNA aptamers specific for transmembrane p24 trafficking protein 6 and Clusterin for the targeted delivery of imaging reagents and RNA therapeutics to human β cells
Source: Nat Commun. 2022 Apr 5;13:1815. doi: 10.1038/s41467-022-29377-3 (PMC8983715; doi:10.1038/s41467-022-29377-3)
Supplement: Supplementary file 3 — Description of Additional Supplementary Information [file 41467_2022_29377_MOESM3_ESM.docx]

**Inventory of Supporting Information**

The supplementary information zip file include:

1. ***The supplementary information pdf*** contains:

**Supplementary figures**

- Supplementary Figure 1: 3D structures of the 15 aptamers chosen for empirical validation
- Supplementary Figure 2: Aptamer binding to human tissue arrays highlights a good specificity of aptamer 1-717 and m12-3773 for human islets
- Supplementary Figure 3: Scanned images of human pancreas sections stained with aptamer 1-717, m12-3773, or scrambled aptamers.
- Supplementary Figure 4. An optimized immune fluorescence staining technique shows that aptamer 1-717 and m12-3773 are highly specific for human islets.
- Supplementary Figure 5: Example of image cytometry analysis.
- Supplementary Figure 6: Affinity of aptamer m12-3773 and 1-717 for human β cells and MIN6 cells.
- Supplementary Figure 7: Cold target inhibition assays indicates TMED6 as putative target for aptamer 1-717.
- Supplementary Figure 8: Aptamer 1-717 and m12-3773 do not show toxicity in vivo or in vitro
- Supplementary Figure 9: Aptamers 1-717 and m12-3773 cross-react with mouse tissues.

**Supplementary Tables:**

- Supplementary table 1: Aptamer selected from the “Human cluster cell SELEX”
- Supplementary table 2: Aptamer selected from the “Toggle cell SELEX”
- Supplementary table 3: primers and aptamer template sequences
- Supplementary table 4: Donor and characteristics of islet preparation.
- Supplementary table 5: proteins isolated via aptamer mediated immune precipitation and mass spectrometry analysis
- Supplementary table 6: candidate saRNAs for human Xiap

1. ***The supplementary data file 1*** is an excel spreadsheet containing the data protein array screening for potential 1-717 binder merged with the data from the differential genechip analysis of genes expressed on the islets over the whole pancreas.
2. **The uncropped figure 3a**
