# Supplementary material for: RNA aptamers specific for transmembrane p24 trafficking protein 6 and Clusterin for the targeted delivery of imaging reagents and RNA therapeutics to human β cells
Source: Nat Commun. 2022 Apr 5;13:1815. doi: 10.1038/s41467-022-29377-3 (PMC8983715; doi:10.1038/s41467-022-29377-3)
Supplement: Supplementary file 4 — Reporting summary [file 41467_2022_29377_MOESM4_ESM.pdf]

nature portfolio

Corresponding author(s): Paolo Serafini

Last updated by author(s): Feb 23, 2022

Reporting Summary

Nature Portfolio wishes to improve the reproducibility of the work that we publish. This form provides structure for consistency and transparency in reporting. For further information on Nature Portfolio policies, see our Editorial Policies and the Editorial Policy Checklist.

Statistics

For all statistical analyses, confirm that the following items are present in the figure legend, table legend, main text, or Methods section.

n/a	Confirmed	
☐	☒	The exact sample size (*n*) for each experimental group/condition, given as a discrete number and unit of measurement
☐	☒	A statement on whether measurements were taken from distinct samples or whether the same sample was measured repeatedly
☐	☒	The statistical test(s) used AND whether they are one- or two-sided<br>*Only common tests should be described solely by name; describe more complex techniques in the Methods section.*
☐	☒	A description of all covariates tested
☐	☒	A description of any assumptions or corrections, such as tests of normality and adjustment for multiple comparisons
☐	☒	A full description of the statistical parameters including central tendency (e.g. means) or other basic estimates (e.g. regression coefficient) AND variation (e.g. standard deviation) or associated estimates of uncertainty (e.g. confidence intervals)
☐	☒	For null hypothesis testing, the test statistic (e.g. *F*, *t*, *r*) with confidence intervals, effect sizes, degrees of freedom and *P* value noted<br>*Give P values as exact values whenever suitable.*
☒	☐	For Bayesian analysis, information on the choice of priors and Markov chain Monte Carlo settings
☒	☐	For hierarchical and complex designs, identification of the appropriate level for tests and full reporting of outcomes
☐	☒	Estimates of effect sizes (e.g. Cohen's *d*, Pearson's *r*), indicating how they were calculated

Our web collection on statistics for biologists contains articles on many of the points above.

Software and code

Policy information about availability of computer code

Data collection	Flow cytometry data were acquired with the FACS DIVA v 8.0.1 or Cytexpert v2.3<br>Image scanner images were acquired with Olyvia software v ASW-L100<br>Confocal microscopy images were acquired with Leica LAF v2.7.7.12402<br>protein chip signals were acquired with GenePix Pro Microarray Analysis Software
Data analysis	affy package v. 1.56<br>Aptani v 1 (Caroli et al.,2015) http://aptani.unimore.it/<br>Blast https://blast.ncbi.nlm.nih.gov<br>Blat http://genome.ucsc.edu/cgi-bin/hgBlat<br>Cell profiler v3.1.9 https://cellprofiler.org/<br>Clustal omega v1.2.2 http://www.clustal.org<br>FCS express plus v6 and v7 (Denovo) https://www.denovosoftware.com/<br>HiSeq's Real Time Analysis (RTA) v2 Casava software<br>Imagej v1.53f51 https://imagej.nih.gov/ij/<br>Jalview 2.11.0 https://www.jalview.org/<br>limma package v. 3.34.9<br>Living Image v4.3 software (Perkin Elmer)<br>Mascot software v2.6.00<br>Ms Excel v 2201<br>PAA package v. 1.0<br>saRNA screening algorithm v1 (Wang et al., 2015)

Sigmaplot v12.5 https://www.sigmaplot.com
siRNA wizard invivogen https://www.invivogen.com/sirnawizard/siRNA.php

For manuscripts utilizing custom algorithms or software that are central to the research but not yet described in published literature, software must be made available to editors and reviewers. We strongly encourage code deposition in a community repository (e.g. GitHub). See the Nature Portfolio guidelines for submitting code & software for further information.

Data

Policy information about availability of data

All manuscripts must include a data availability statement. This statement should provide the following information, where applicable:

- Accession codes, unique identifiers, or web links for publicly available datasets
- A description of any restrictions on data availability
- For clinical datasets or third party data, please ensure that the statement adheres to our policy

The proteinchip data is available in the Gene Expression Omnibus (GEO) database under accession code GSE162273.
the sequencing data from the SELEX experiments are available in GEO under accession code GSE197262
we used the following GEO datasets GSE2109 and GSE15543, both for the whole pancreas tissues (GSM53046, GSM325790, GSM325838, GSM277701, GSM277726, GSM277736, GSM231922, GSM203675, GSM203703, GSM203761, GSM179781, GSM179869, GSM152744, GSM137958, GSM117645, GSM117647, GSM89045) and for the islets cells (GSM388749, GSM388750, GSM388753, GSM388754, GSM388759, GSM388760, GSM388766, GSM388767).

All relevant data are available within the article and its Supplementary information files or upon request.

Field-specific reporting

Please select the one below that is the best fit for your research. If you are not sure, read the appropriate sections before making your selection.

☒ Life sciences ☐ Behavioural & social sciences ☐ Ecological, evolutionary & environmental sciences

For a reference copy of the document with all sections, see nature.com/documents/nr-reporting-summary-flat.pdf

Life sciences study design

All studies must disclose on these points even when the disclosure is negative.

Sample size	Sample size was chosen by power analysis using effect size determined by pilot experiments. Post-hoc power analysis was performed in Sigmaplot vs12.5
Data exclusions	No data were excluded from any analysis
Replication	In vitro analyses and in vivo experiments were repeated two to five times to ensure reproducible conclusions; the exact number of repetitions is stated in each figure legend. When possible, the same phenomenon was analyzed with two independent techniques to minimize technique specific artifact.
Randomization	Mice were ear-tagged and randomized before and after treatment. In vitro, aliquots of the same samples were randomly allocated to each experimental group.
Blinding	In vivo measurements (i.e. glucose measurement, IVIS) were taken and analyzed by an investigator blinded to experimental group. The investigators performing the in vitro experiment were not blinded to the experimental groups since they were the ones adding the different treatment however, flow data and digitalized images were analyzed by a computer as described in the methods to guarantee objective measurements and experiments were conducted independently by at least two experimentalists with similar results.

Reporting for specific materials, systems and methods

We require information from authors about some types of materials, experimental systems and methods used in many studies. Here, indicate whether each material, system or method listed is relevant to your study. If you are not sure if a list item applies to your research, read the appropriate section before selecting a response.

Materials & experimental systems

n/a	Involved in the study	
	x	Antibodies
	x	Eukaryotic cell lines
x		Palaeontology and archaeology
	x	Animals and other organisms
	x	Human research participants
x		Clinical data
x		Dual use research of concern

Methods

n/a	Involved in the study	
x		ChIP-seq
	x	Flow cytometry
x		MRI-based neuroimaging

Antibodies

Antibodies used	Guinea pig anti-Insulin (1:300) DAKO Cat# A0564; RRID: AB_2617169;<br>Rabbit anti-Glucagon (1:300) Cell Signaling Cat# 2760; RRID: AB_659831;<br>Anti-Insulin APC (1:10) R&D Systems Cat# IC1417A, RRID:AB_2126535;<br>Anti-glucagon PB (1:100) BD Biosciences Cat# 565860, RRID: AB_2739382;<br>Goat anti-Rabbit IgG, AF 488 (1:400) Thermo Fisher Scientific Cat# A-11034, RRID:AB_2576217;<br>Goat anti-GuineaPig IgG AF647 (1:400) Thermo Fisher Scientific Cat# A-21450, RRID:AB_2735091;<br>5'Biotin- Apt#1-717, Apt#m12-3773, APTSCR1-717, and APTSCRm12-3773 Oligofactory Custom order (suppl. table 3);
Validation	Commercial antibodies validated by each manufacturer have been titrated and each lot checked for consistency with previous ones using serial sections or technical replicate in flow cytometry.

Eukaryotic cell lines

Policy information about cell lines

Cell line source(s)	A549 and MIN6 were purchased from ATCC
Authentication	A549 and MIN6 were not authenticated
Mycoplasma contamination	all cell lines were tested negative for mycoplasm and other common pathogen contamination by external vendor via RT-PCR
Commonly misidentified lines<br>(See ICLAC register)	none

Animals and other organisms

Policy information about studies involving animals; ARRIVE guidelines recommended for reporting animal research

Laboratory animals	All animal experiments were approved by the Division of Veterinary Resources and the Institutional Animal Care & Use Committee of the University of Miami. Eight to ten weeks old male NOD.Cg-Prkdcscid Il2rgtm1Sug/JicTac (NOG) (Taconic), female Balb/C, and C57Bl/6J mice (Jackson) were purchased, allowed free access to food and water and were maintained on a 12-h light/dark cycle at room temperature (range 20-23°C) and controlled humidity (range 30-70%) in in individually ventilated cages at the pathogen free animal facilities at the University of Miami on a chlorophyll free diet.
Wild animals	no wild animals were used in the study.
Field-collected samples	no field collected samples were used in the study.
Ethics oversight	All animal experiments were approved by the Division of Veterinary Resources and the Institutional Animal Care & Use Committee of the University of Miami.

Note that full information on the approval of the study protocol must also be provided in the manuscript.

Human research participants

Policy information about studies involving human research participants

Population characteristics	No human participant were involved in the study, however, we used de-identified tissues from cadaveric donors. This study is not a human study as defined by NIH. Tissue and donor information are reported in supplementary table 4. Cadaveric donors were non-diabetic with a median age of 49 years (range 23-63), median BMI 27.5 (IQ range 18.2-33). 31% were black, 25% white not hispanic, and 44% white hispanic. 50% of the cadaveric donors were male and 50% female
Recruitment	de-identified islets were acquired through commercial vendors or through the GMP facility at the Diabetes research institute

Ethics oversight	Ethics oversight was not needed in this study since the study used only tissues from de-identified cadaveric donors collected by commercial vendors or by the GMP facility at the DRI. As such this study is not considered a human stucy as per NIH definition and thus does not require ethycal oversight.

Note that full information on the approval of the study protocol must also be provided in the manuscript.

Flow Cytometry

Plots

Confirm that:

- [x] The axis labels state the marker and fluorochrome used (e.g. CD4-FITC).
- [x] The axis scales are clearly visible. Include numbers along axes only for bottom left plot of group (a 'group' is an analysis of identical markers).
- [x] All plots are contour plots with outliers or pseudocolor plots.
- [x] A numerical value for number of cells or percentage (with statistics) is provided.

Methodology

Sample preparation	Islet cultures were spun down at 250g for 6 min. The supernatant was decanted, and islets incubated with 400µl of 4ºC trypsin for 5-10 min'. Reaction was quenched with 20% FBS containing RPMI. Islets were passed 5 times through a 5/8 26G needle, and cells spun at 500g for 6.5 min and washed with PBS. Cells, were stained with the fixable Live Dead dye (Live/dead yellow, ThermoFisher) for 20 min at RT, washed and incubated for 30 min at 4ºC with cy3-labelled RNA aptamers (38 pmoles/ 250IEQ). The cell suspension was then washed once in PBS, spun at 550g for 6.5 min, permeabilized and fixed with the 1 Perm/fix solution (BD Bioscience) for 20 min at 4ºC, washed twice with 1x perm/wash buffer, and stained with anti-insulin and anti-glucagon antibodies for 30 min at 4ºC. MIN6 cells were detached with trypsin and incubated for 30' at 4C with either aptamer chimera hybridized to Cy5 guide RNA or biotinylated aptamer complexed with streptavidin-AF647 in HBBS. Samples were washed once again with perm/wash buffer and once with PBS, resuspended in 300 µl of PBS. Data were acquired with FACS DIVA v 8.0.1 or Cytexpert v2.3 software and analyzed using FCS v6 and v7 express plus software (Denovo-Software).
Instrument	Samples were analyzed on a LSR2 flow cytometer equipped with 405nm, 488nm, 532nm, and 635nm lasers (BD Bioscience) or a cytoflex 18.
Software	Data were acquired with FACS DIVA v 8.0.1 or Cytexpert v2.3 software and analyzed using FCS v6 and v7 express plus software (Denovo-Software).
Cell population abundance	no sorting experiments were performed in this study.
Gating strategy	relevant gating strategies are depicted in the figures or in the supplementary data. Positive and negative populations were determined using FMO controls

- [x] Tick this box to confirm that a figure exemplifying the gating strategy is provided in the Supplementary Information.
